# The crystalline state as a dynamic system: IR microspectroscopy under electrochemical control for a [NiFe] hydrogenase†

**DOI:** 10.1039/d1sc01734a

**Published:** 2021-06-03

**Authors:** Philip A. Ash, Sophie E. T. Kendall-Price, Rhiannon M. Evans, Stephen B. Carr, Amelia R. Brasnett, Simone Morra, Jack S. Rowbotham, Ricardo Hidalgo, Adam J. Healy, Gianfelice Cinque, Mark D. Frogley, Fraser A. Armstrong, Kylie A. Vincent

**Affiliations:** Department of Chemistry, University of Oxford, Inorganic Chemistry Laboratory South Parks Road Oxford OX1 3QR UK philip.ash@leicester.ac.uk kylie.vincent@chem.ox.ac.uk; School of Chemistry, University of Leicester Leicester LE1 7RH UK; Leicester Institute of Structural and Chemical Biology, University of Leicester LE1 7RH UK; Research Complex at Harwell, Rutherford Appleton Laboratory, Harwell Campus Didcot UK; Diamond Light Source, Harwell Science and Innovation Campus Didcot OX11 0QX UK; Department of Engineering Sciences, University of Oxford Parks Road Oxford OX1 3PJ UK

## Abstract

Controlled formation of catalytically-relevant states within crystals of complex metalloenzymes represents a significant challenge to structure–function studies. Here we show how electrochemical control over single crystals of [NiFe] hydrogenase 1 (Hyd1) from *Escherichia coli* makes it possible to navigate through the full array of active site states previously observed in solution. Electrochemical control is combined with synchrotron infrared microspectroscopy, which enables us to measure high signal-to-noise IR spectra *in situ* from a small area of crystal. The output reports on active site speciation *via* the vibrational stretching band positions of the endogenous CO and CN^−^ ligands at the hydrogenase active site. Variation of pH further demonstrates how equilibria between catalytically-relevant protonation states can be deliberately perturbed in the crystals, generating a map of electrochemical potential and pH conditions which lead to enrichment of specific states. Comparison of in crystallo redox titrations with measurements in solution or of electrode-immobilised Hyd1 confirms the integrity of the proton transfer and redox environment around the active site of the enzyme in crystals. Slowed proton-transfer equilibria in the hydrogenase in crystallo reveals transitions which are only usually observable by ultrafast methods in solution. This study therefore demonstrates the possibilities of electrochemical control over single metalloenzyme crystals in stabilising specific states for further study, and extends mechanistic understanding of proton transfer during the [NiFe] hydrogenase catalytic cycle.

## Abstract 2

Electrochemically-coupled IR microspectroscopy of single crystals provides insight into proton-coupled electron transfer in [NiFe] hydrogenase.

## Graphical abstract

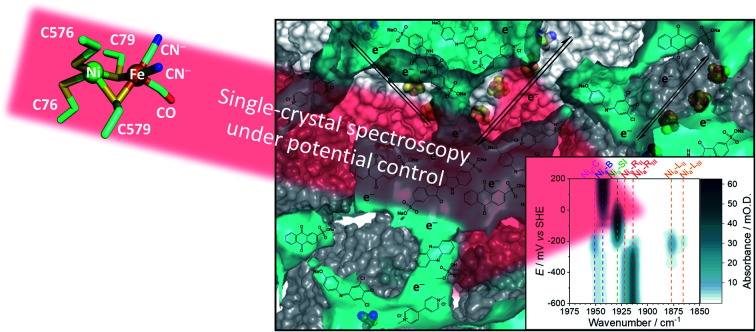

## Introduction

Obtaining crystals of redox enzymes in intermediate states relevant to catalysis is a high-profile, yet challenging target. Methods for controlling the redox state of protein crystals include the titration of crystal medium with reductant or oxidant until a desired solution potential is reached,^1,2^ exposure of crystals to substrate/inhibitor,^3,4^ or crystallisation of protein which has been pre-equilibrated to a desired redox state.^5,6^ However, these methods often lack precision in generating pure enzyme states. There is also growing interest in triggering catalytic steps in enzyme crystals which can be coupled with time-resolved serial synchrotron or XFEL crystallography, and to date, such methods have typically relied on photo-triggers for reactivity.^7–9^ Methods for studying crystalline and lyophilised enzyme using gas exchange have also been reported.^10^ Verification of protein redox states in crystallo presents a further challenge, and to this end, a number of synchrotron macromolecular crystallography beamlines have introduced microspectroscopic methods for secondary characterisation of protein crystals, including UV-visible and Raman spectroscopy.^11–13^

We have previously demonstrated the possibility of electrochemical control over single crystals of hydrogenase I from *Escherichia coli* (Hyd1) coupled with synchrotron infrared (IR) microspectroscopy for simultaneous reporting on the active site speciation.^14^ Vibrational absorption bands of the integral CO and CN^−^ ligands at the active site of hydrogenases make this spectroscopic method ideal for elucidating the redox and coordination state of the active site. By applying steps in electrode potential, we were able to achieve uniform and reversible manipulation of Hyd1 in crystallo from the most oxidised to the most reduced levels. We now show how fine potential control over Hyd1 crystals can be used to generate specific redox levels, enabling us to control and examine transitions between catalytically-relevant redox and protonation states.

**Scheme 1:**
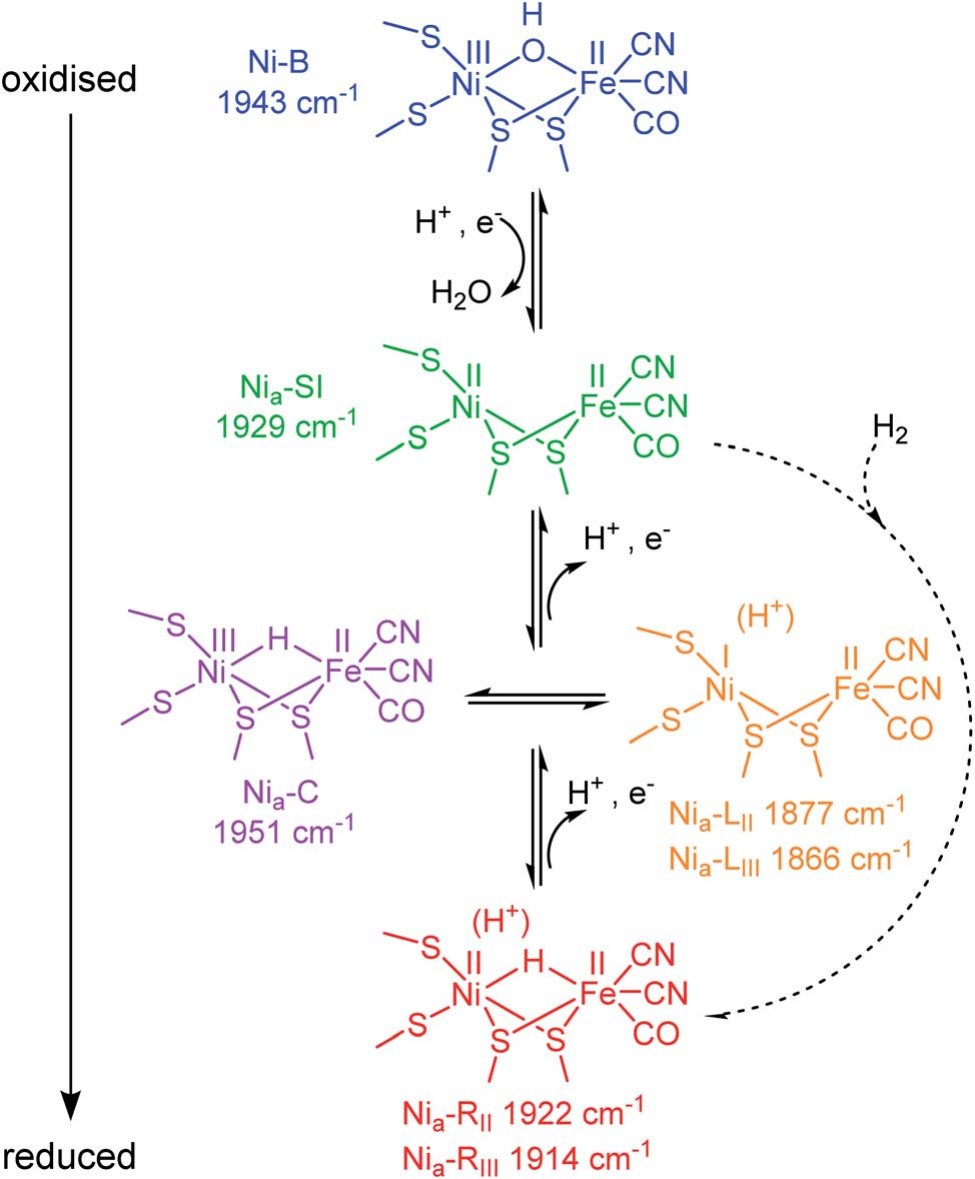
Skeletal structure of the active site redox states for [NiFe] hydrogenases ordered by redox level. Dashed arrows represent the H_2_ binding and activation step during catalytic H_2_ oxidation. States are colour-coded to match data throughout this work. Catalytically active states are labelled “Ni_a_–X”, where X = SI, C, L or R. *ν*_CO_ band positions refer to Hyd1, pH 5.9.

Hydrogenases are a broad group of enzymes responsible for bidirectional heterolytic activation of dihydrogen (H_2_ ⇌ H^+^ + H^−^ → 2H^+^ + 2e^−^) at di-iron or nickel–iron bimetallic active sites.^15,16^ They have attracted attention for wide-ranging applications in biotechnology: energy applications in microbial H_2_ production and bioanodes for H_2_/O_2_ fuel cells, through to H_2_-driven biocatalytic cascades.^17–21^ The active sites of most hydrogenases are ‘wired’ to a bacterial membrane or to their natural redox partner *via* a chain of FeS clusters in the protein. In the [NiFe] enzymes, the active site Ni atom is ligated by two terminal cysteine thiolates, with two additional cysteines bridging to the Fe atom. The Fe is further coordinated by one CO and two CN^−^ ligands (Scheme 1). During catalysis, the Ni formally cycles through Ni^I/II/III^, whereas the Fe remains formally Fe^II^, presumably stabilised by buffering of electron density from the combination of pi-acceptor and sigma-donor properties of the coordinated CO and CN^−^ ligands.^16^ The CO and CN^−^ stretching bands in the mid-IR (*ν*_CO_ and *ν*_CN_ respectively) respond sensitively in wavenumber position to changes in electron density at the active site, and even to protonation and changes in hydrogen-bonding in the vicinity of the active site.^22^ Since protons (H^+^) are the product/substrate of hydrogenases, the activity and speciation of these enzymes are greatly pH-dependent.^23,24^ It is possible, electrochemically, to step through the range of catalytically-relevant redox levels of hydrogenase by controlling the electron transfer and proton-coupled electron transfer (PCET) steps, as shown in Scheme 1.

A catalytic cycle for [NiFe] hydrogenases has been proposed by combining insight from spectroscopic, computational, structural and activity studies.^16^ Viewed in the direction of H_2_ oxidation (Scheme S1†), it is generally accepted that H_2_ binds at the Ni^II^ redox level, ‘Ni_a_–SI’, the most oxidised catalytic state (subscript ‘a’ denoting an ‘active’ catalytic species), however, any Michaelis complex with H_2_ has evaded detection to date. Heterolytic cleavage of H_2_ leaves a hydridic H in a bridging position between the Ni and Fe,^25^ and a proton on a nearby base, the identity of which remains hotly debated.^26–28^ The resulting state is generally termed Ni_a_–R, though several Ni_a_–R sub-states exist, likely reflecting sequential proton movement away from the active site.^22^ An electron must next be transferred from the active site to the FeS cluster relay chain to form the Ni_a_–C state which still contains a bridging hydride, but is formally Ni^III^.^29–31^ From the Ni_a_–C state, the bridging hydride is lost as a proton to a nearby base (not necessarily the same base that accepts the initial proton from H_2_ cleavage at the Ni_a_–R level^32^) leaving the Ni reduced formally by two electrons to Ni^I^ (generally termed ‘Ni_a_–L’, noting that there are also multiple Ni_a_–L sub-states, again likely reflecting differential proton location in the region of the active site). Since the transition from Ni_a_–C to Ni_a_–L simply requires relocation of electron density and a proton, these two states have been described as tautomeric and exist at the same redox level (Scheme 1). Although Ni_a_–L was first observed as a low temperature photo-product of Ni_a_–C, evidence for Ni_a_–L as a catalytic intermediate has accumulated from a series of steady state and transient spectroscopic studies which support earlier theoretical mechanistic proposals.^22,33–37^ Finally, the Ni must be oxidised back to Ni^II^*via* electron transfer to the FeS cluster chain, to re-generate the Ni_a_–SI state ready to bind the next molecule of H_2_.

Hyd1 belongs to the group of so-called O_2_-tolerant hydrogenases, along with membrane bound hydrogenase (MBH) enzymes from *Ralstonia eutropha* (*Cupriavidus necator*), *Aquifex aeolicus*, and *Hydrogenovibrio marinus*.^15^ Hydrogenases within this group differ principally in the structure and potential of the electron-relay FeS cluster proximal to their active site, and the unusually high potential of this cluster has been linked to their O_2_-tolerance.^38–42^

Like other [NiFe] hydrogenases, Hyd1 is isolated in a mixture of oxidised inactive states that require reductive activation.^43,44^ The predominant state is Ni–B, which has a bridging OH^−^ ligand ^16,45^ (Scheme 1), and is reversibly re-formed following oxidation, particularly at low H_2_.^46^

Despite a wealth of spectroscopic, structural, and biophysical studies on hydrogenases from diverse organisms, many details of the mechanism of H_2_ activation remain uncertain. Individual proton and electron transfer events, how they are temporally linked to the catalytic mechanism and/or formation and reactivation of inactive states, the identities of proton donors and acceptors, and the identity of key catalytic intermediates are all questions that remain unanswered for both the [NiFe] and [FeFe] hydrogenases.

One of the challenges is how to unify the understanding gained from measurements made on different physical sample types. Spectroscopy of hydrogenases is typically performed in solution, and solution IR spectroelectrochemical ‘redox titrations’ are well established for hydrogenases, with use of small-molecule redox mediators to facilitate diffusion-controlled electron transfer.^16^ Frozen samples are required for nuclear resonance vibrational spectroscopy and most EPR measurements, while crystalline material is required for structure determination. Electrochemistry on films of electrode-immobilised protein (Protein Film Electrochemistry, PFE) has been used widely in studying hydrogenases,^47–49^ and we have previously introduced a complementary IR spectroelectrochemical approach termed Protein Film Infrared Electrochemistry (PFIRE) which provides chemical/structural insight to complement information from PFE alone.^32,50,51^

Here, we compare the potential-dependence of IR-detected equilibrium active site states observed for Hyd1 under electrochemical control in solution, on an electrode (PFIRE) and in single crystals. Significantly, we now show that, within single crystals of Hyd1, it is possible to achieve control over the full manifold of states observed in solution for this enzyme. A related report by Morra *et al.* demonstrates electrochemical manipulation of [FeFe] hydrogenase I from *Clostridium pasteurianum* using similar methods,^78^ and these studies present the possibility for using electrochemical control over single protein crystals to establish samples in the solid state for further structural study.

## Experimental methods

### Purification of Hyd1

Hyd1 was prepared aerobically according to a published procedure.^52^ For PFIRE and solution-based experiments, no further purification was required, however for crystallisation it was essential to remove any aggregated protein and bound cytochrome-b subunit *via* size exclusion chromatography (SEC) followed by hydroxyapatite chromatography, as described previously.^26^ Fractions containing highly pure Hyd1 (HyaAB) were identified by SDS-PAGE, pooled and buffer exchanged into SEC buffer (20 mM Tris, pH 7.2, 150 mM NaCl, 0.02% (w/v) DDM detergent, 1 mM dithiothreitol) by repeated spin concentration and dilution (using Vivaspin 20 mL, 50 kDa molecular weight cut-off centrifugal concentrators until a 1500–2000 fold dilution of the phosphate buffer used during hydroxyapatite chromatography was achieved). For crystal growth, protein samples were concentrated to 5 mg mL^−1^, as judged by Bradford assay.^53^ Crystals of Hyd1 were acquired according to previously established protocols,^26^ using the sitting drop vapour diffusion technique, where 1.5 μL of protein solution was mixed with an equal amount of crystallisation buffer (either 100 mM Bis–Tris, pH 5.5–5.9, 200 mM Li_2_SO_4_, 150 mM NaCl, PEG 3350 (19–21% w/v) or 100 mM Tris·HCl, pH 8.0, 200 mM Li_2_SO_4_, 150 mM NaCl, 19–21% PEG 3350) followed by streak seeding with old smaller crystals of Hyd1. Incubation under an anaerobic atmosphere (<0.3 ppm O_2_) at 20 °C resulted in crystals appearing within 24 hours.

### Single-crystal IR microspectroscopic-electrochemical experiments

An adaptation of our previously-reported cell design^14,54^ was used for single-crystal microspectroscopic electrochemistry, and is described in more detail in the ESI (Fig. S1†). The microspectroscopic-electrochemical cell contained a miniature Ag/AgCl reference electrode (3 M KCl, 2 mm diameter, eDAQ), a graphite ring counter electrode (cut from a graphite tube, Goodfellow), and a glassy carbon working electrode (4 mm diameter, Alfa Aesar). The working electrode was polished to high reflectivity (*ca.* 10–20% in the mid-IR) using increasingly fine grades of silicon carbide paper (2500 and 4000 grit, Kemet). The polished electrodes were washed by ultrasonication in ultrahigh purity water (MilliQ, 18 MΩ cm) prior to cell assembly. The reference electrode was removed during the polishing process to avoid damage and contamination.

A solution containing the redox mediators 2,6-dichloroindophenol, phenazine methosulfate, indigo carmine, anthroquinone-2-sulfonate, and methyl viologen, each at 1 mM concentration (Table S1†) was prepared in N_2_-degassed crystal stabilisation buffer (for experiments conducted at pH 5.9 this was 100 mM Bis–Tris, pH 5.9, 200 mM Li_2_SO_4_, 150 mM NaCl, 22% v/v PEG 3350, whereas for experiments conducted at pH 8.0 the buffer used was 100 mM Tris, pH 8.0, 200 mM MgCl_2_, 150 mM NaCl, 22% PEG 3350). A 3 μL aliquot of the mediator solution was added to each well of a crystallisation plate containing Hyd1 crystals (crystals were stored in ∼3 μL mother liquor, and the size and number of crystals varied between wells). Gentle pipetting suspended the crystals without damaging crystal integrity. The resulting 6 μL mixture containing redox mediators and Hyd1 crystals was then deposited onto the glassy carbon working electrode of the microspectroscopic-electrochemical cell. An additional 12 μL of redox mediator solution in crystal stabilisation buffer was then pipetted onto the Ag/AgCl reference electrode and graphite counter electrode such that the cell was filled with approximately 18 μL of *ca.* 0.66 mM mediator solution. A CaF_2_ window (UV grade, 30 mm diameter, 1 mm thickness, Crystran) was sealed onto the cell surface using a PTFE gasket (Harrick, 25 μm thick) and silicone sealant (Dowsil, SE 9187L Silicone RTV) to maintain an anaerobic environment within the cell. Assembly of the IR microspectroscopic-electrochemical cell, crystal handling, and mediator solution preparation, were carried out in a N_2_-filled glovebox (Plas-Labs Inc., 815 PGB series, <20 ppm O_2_). The addition of redox mediators facilitates diffusion-controlled transfer of electrons through the electrolyte to enable electron transfer between the working electrode and the crystalline protein (a representative cyclic voltammogram of the mediator solution is shown in Fig. S2†). Solvent channels within the Hyd1 crystal have radii between 5.4–6.6 Å (calculated using pdb 6FPO and MAP_CHANNELS^55^) and are thus large enough to allow diffusion of redox mediators throughout the crystal (Fig. S3†).

IR microspectroscopic-electrochemical experiments were carried out on the MIRIAM beamline B22 at Diamond Light Source, UK, using a Vertex 80V Fourier transform IR spectrometer coupled to a Hyperion 3000 IR microscope (Bruker) with a high-sensitivity photovoltaic mercury cadmium telluride (MCT) 50 μm pitch detector cooled to 77 K using liquid N_2_. A transflection geometry was used to obtain IR spectra (*i.e.* the microscope is used in reflection mode and detected light that passes through the cell twice), using a 36× objective and 15 × 15 μm^2^ knife-edge aperture in the detection beampath. Each spectrum was recorded as an average of 1024 interferograms working at 80 kHz scanner velocity and at 4 cm^−1^ resolution (*ca.* 160 s measurement time). Data acquisition was performed using Bruker OPUS software (version 7.5). Electrochemical measurements were acquired using an AutoLab 128N potentiostat (Metrohm) controlled by Nova software (version 1.10). The miniature Ag/AgCl reference electrode was calibrated against a saturated calomel reference electrode (SCE, BAS), and potentials quoted in the text are adjusted to mV *vs.* the standard hydrogen electrode (SHE) using the conversion *E* (mV *vs.* SHE) = *E* (mV *vs.* SCE) + 241 mV at 25 °C.^56^ Baseline correction, and all subsequent data analysis was carried out using OriginPro software (OriginLab Corp., version 9.1). Baseline correction was applied using an interpolated spline function, and careful comparison with 2^nd^ derivative and difference spectra was used to avoid distortion of peak shapes. Baseline corrected spectra are presented in the main text, and representative raw spectra are shown in the ESI.†

### Solution infrared spectroscopic-electrochemical measurements

Electrochemically-controlled IR redox titrations of solution phase Hyd1 were recorded using our previously-reported methods.^57,58^ Briefly, a 3D carbon particle network electrode^59^ containing Hyd1 trapped within a mixture of the polymer electrolyte Nafion (Sigma, titrated to pH 6 in phosphate buffer) and carbon black particles (XC72R, DUPONT) was prepared on the surface of an ATR-IR accessory (GladiATR, Pike Technologies) housed in an anaerobic, dry glovebox (Glove Box Technologies). The 3D network electrode was sealed into an electrochemical cell containing a carbon rod working electrode connection, saturated calomel reference electrode, and a Pt wire counter electrode. A closed loop of N_2_-purged electrolyte was pumped through the cell to prevent build-up of any trace H_2_ produced by Hyd1. For more details see ESI.†

IR spectra were recorded using an Agilent 680-IR spectrometer controlled by ResPro 4 software, as an average of 1024 interferograms (*ca.* 360 s measurement time). Electrochemical control was provided by an Autolab 128N potentiostat (Metrohm), and potentials (*E*) are reported relative to SHE using the conversion *E* (mV *vs.* SHE) = *E* (mV *vs.* SCE) + 241 mV at 25 °C.^56^

### Infrared spectroscopic-electrochemical measurements of electrode-adsorbed Hyd1 (PFIRE)

The IR spectroscopic data collected from electrode-adsorbed Hyd1 are reproduced using data from Hidalgo *et al.*^51^ The PFIRE method is briefly described in the ESI.† In order to aid comparison to our single crystal method, we report the absorbance of individual Ni_a_–R sub-states separately in this manuscript, whereas they were summed to give a ‘total’ Ni_a_–R absorbance in Hidalgo *et al.* We have also reassigned some of the Ni_a_–L absorbances relative to the original manuscript such that both the Ni_a_–L_I,II,III_ and Ni_a_–R_I,II,III_ sub-states are labelled in order of decreasing wavenumber of the active site CO stretch, *ν*_CO_, in line with other literature.^35,36,60–66^

## Results and discussion

### Initial characterisation and electrochemical reduction of crystalline Hyd1

**Fig. 1:**
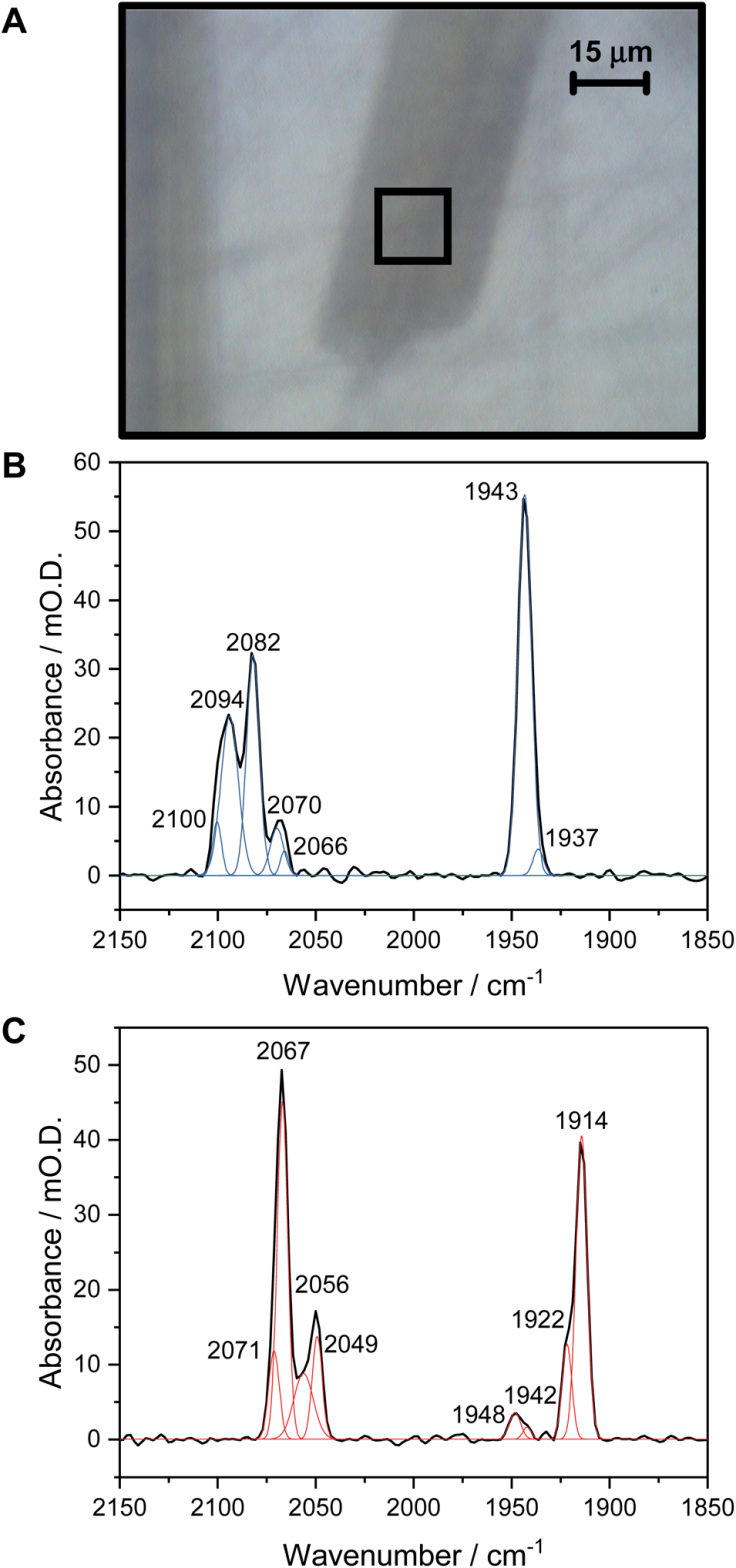
(A) Visible image at 36× magnification of a single Hyd1 crystal on the working electrode of the IR microspectroscopic-electrochemical cell, showing the 15 × 15 μm^2^ sampling area used to collect IR spectra. (B) IR spectrum collected on a single crystal prepared from as-isolated Hyd1 at pH 5.9, equilibrated at open circuit potential (+209 mV), showing the *ν*_CO_ and *ν*_CN_ regions. (C) IR spectrum collected on a Hyd1 single crystal at pH 5.9 at an applied potential of −597 mV after reduction for 1 hour.

Fig. 1A shows a visible image, at 36× magnification, of a single Hyd1 crystal lying on the working electrode surface (fine scratches are also visible in the glassy carbon surface, and another crystal can be seen to the left of the image, roughly vertically oriented). The 15 × 15 μm^2^ area used to record IR spectra is shown with a black square. Prior to electrochemical manipulation of the crystal, an IR spectrum was recorded at the open circuit potential (OCP) imposed by the oxidised mediator cocktail (typical OCP values were +209 to +274 mV *vs.* SHE). Fig. 1B shows a representative IR spectrum of crystalline Hyd1 recorded at pH 5.9 and an OCP of +209 mV before any electrochemical manipulation, showing the CN^−^ and CO stretching regions, *ν*_CN_ and *ν*_CO_, respectively (for raw data see Fig. S4†). Crystals prepared from aerobically purified ‘as-isolated’ Hyd1 contain a mixture of oxidised inactive states as is common for [NiFe] hydrogenases,^16^ predominantly the Ni–B state (Fig. 1B, *ν*_CO_ 1943 cm^−1^) with minor contributions from another oxidised species with *ν*_CO_ 1937 cm^−1^. The identity of this minor component is unknown, but similar species have been observed in other hydrogenases and attributed to readily-activated species at the same redox level as Ni_a_–SI.^16,22^ Very intense absorbances are observed from crystalline Hyd1 due to the high effective protein concentration within the crystals (*ca.* 8 mM of active site, see ESI†). Furthermore, the transflection geometry of the microspectroscopic-electrochemical cell means that the effective IR pathlength through the sample is of the order of 30–50 μm, approximately double the crystal thickness. In the case of the crystal sample shown in Fig. 1A we can estimate the molar extinction coefficient of the Ni–B *ν*_CO_ band as approximately 4000 M^−1^cm^−1^, in good agreement with the extinction coefficient reported for this state of the active site in the large subunit of *Ralstonia eutropha* MBH in solution.^67^

Crystalline Hyd1 was subjected to electrochemical reduction by applying a potential of −597 mV for a minimum of 1 hour, until no further changes in *ν*_CO_ and *ν*_CN_ bands were observed over a period of 10 minutes. Electrochemical reduction of crystalline Hyd1 is somewhat analogous to reductive activation used in PFE and PFIRE measurements on [NiFe] hydrogenase,^32,68^ and as shown in Fig. 1C resulted in crystals containing Hyd1 in a mixture of the most reduced Ni_a_–R states, predominantly Ni_a_–R_II_, 1922 cm^−1^, and Ni_a_–R_III_, 1914 cm^−1^ (alternatively referred to in the literature as Ni–SR′ and Ni–SR′′ respectively, see Horch *et al.*, for example^64^). A small contribution from a species with *ν*_CO_ at 1948 cm^−1^ could reflect either a small population of Ni_a_–R_I_ or a Ni_a_–C-like species but this is unclear. Ni–B is not observed after reductive treatment of the crystal confirming that all the crystalline Hyd1 responds to the applied potential (for raw data see Fig. S5†).

### Potential-controlled single-crystal redox titration of Hyd1, pH 5.9, monitored by IR microspectroscopic electrochemistry

**Fig. 2:**
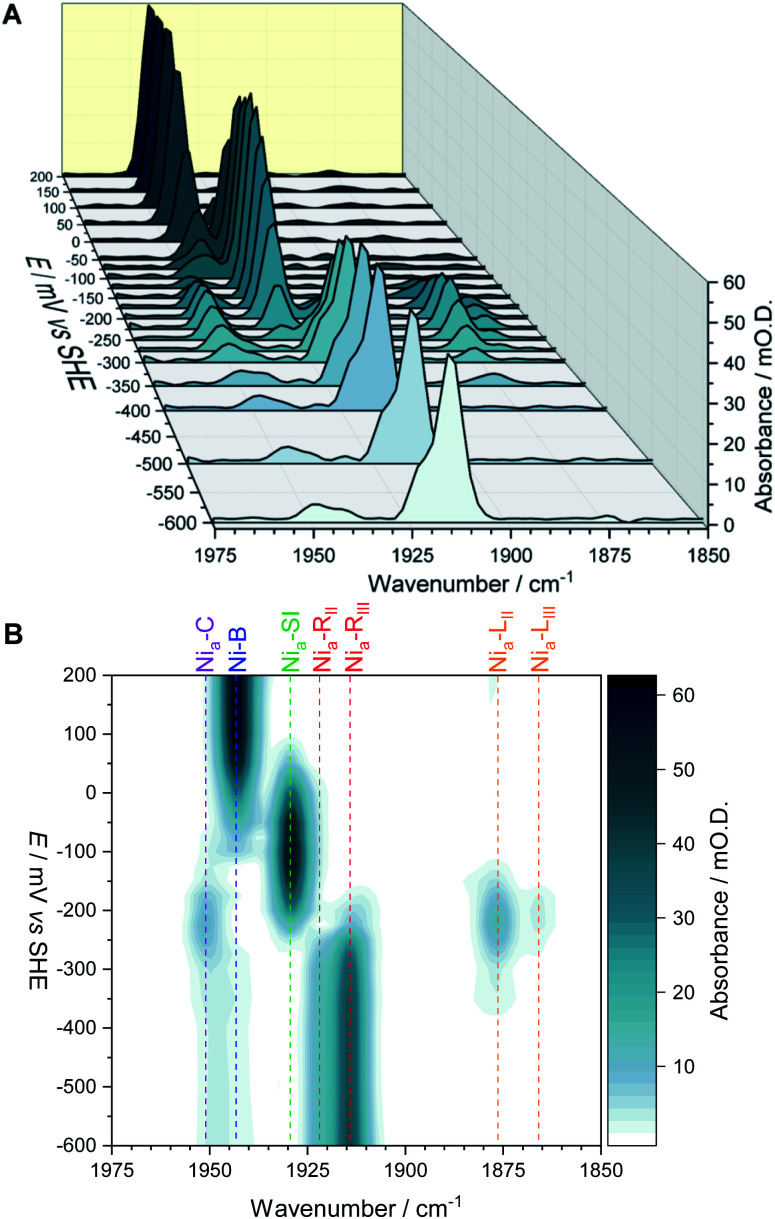
The *ν*_CO_ region observed during electrochemical oxidative titration of a single crystal of Hyd1 at pH 5.9 recorded using the IR microspectroscopic-electrochemical technique showing the potential dependence of each active site redox species. (A) Baseline corrected IR spectra of *ν*_CO_ region. (B) Heatmap of *ν*_CO_ region.

**Fig. 3:**
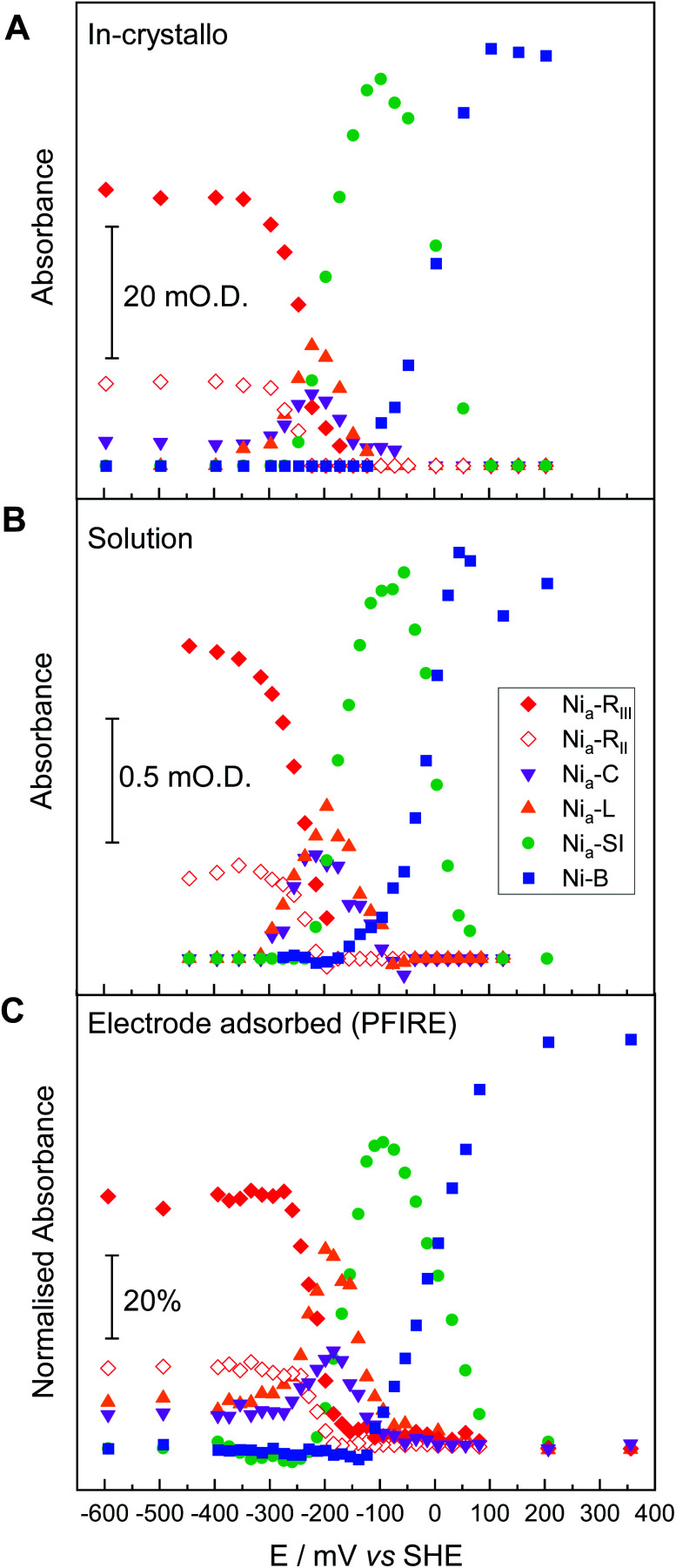
Comparison of redox speciation curves (from oxidative titrations) of Hyd1 at pH 5.9 by three IR spectroscopic-electrochemical methods: single crystal IR microspectroscopic electrochemistry/in crystallo (A); in solution (B); and PFIRE/electrode adsorbed (C). The relative band positions in cm^−1^ for each species per methodology are provided in Table S2.† For clarity the intensities arising from the Ni_a_–L_II_ and Ni_a_–L_III_ species have been summed and are represented collectively as Ni_a_–L here.

The fine potential control afforded by the microspectroscopic-electrochemical cell and redox mediator mixture enables access within the crystal to the intermediate oxidation states of Hyd1 shown in Scheme 1. Fig. 2 shows a series of spectra of a single Hyd1 crystal recorded as a function of applied potential at pH 5.9, as both baseline-corrected spectra (Fig. 2A) and a 2D ‘heatmap’ plot (Fig. 2B). Fig. 2 focusses on the *ν*_CO_ region only, data including the *ν*_CN_ region are shown in Fig. S6,† and raw data are shown in Fig. S7.† These spectroscopic data were recorded following electrochemical reduction at −597 mV, as a series of small steps (25–100 mV per step) towards more positive potentials were applied between −597 mV and +203 mV. After each step the potential was held until spectroscopic equilibration was achieved, as judged by no further changes to IR spectra (or a minimum of 8.5 minutes; corresponding chronoamperometry data shown in Fig. S8†). The changes in Hyd1 active site speciation during this in crystallo oxidative redox titration can be seen from the potential-dependent shift in *ν*_CO_ bands in Fig. 2. These correlate well with the ladder of redox states shown in Scheme 1. The Ni_a_–R species (1922 and 1914 cm^−1^) dominate at the most reducing potentials, converting to the Ni_a_–C (1951 cm^−1^) and Ni_a_–L (1877 and 1866 cm ^−1^) states at intermediate potentials, and then forming the most oxidised catalytically active state Ni_a_–SI (1929 cm^−1^, maximum intensity at −122 mV). At the most oxidising potentials the oxidised, inactive state Ni–B dominates (1943 cm^−1^). Significantly, all previously established active site states for Hyd1 are observed in crystallo, including multiple sub-states of Ni_a_–R and Ni_a_–L. Fitting of the *ν*_CO_ bands to Gaussian band profiles and extracting the fitted peak absorbances allows plotting of titration curves of each active site state as a function of potential, as shown in Fig. 3A (representative spectral peak fitting shown in Fig. S9†).

In order to correlate states observed in Hyd1 crystals with the potential dependence of states observed in more conventional spectroscopic-electrochemical studies, Fig. 3 compares equilibrium potential-controlled redox titrations of the Hyd1 active site in single crystals (Fig. 3A), of Hyd1 in solution (Fig. 3B) and of electrode-adsorbed Hyd1 (Fig. 3C, recorded under an Ar atmosphere, reproduced using data from Hidalgo *et al.*).^51^ The assignments of the *ν*_CO_ and *ν*_CN_ bands for each active site redox species in the crystalline state are consistent with those observed in both solution and electrode-adsorbed IR spectra of Hyd1 (Table S2†). At pH 5.9, there is little or no catalytic H^+^ reduction by Hyd1 (ref. 24 and 51) and as such the data in Fig. 3, recorded under an inert atmosphere, reflect essentially non-turnover behaviour of the Hyd1 active site. The titration curves measured from crystalline, solution, and electrode-adsorbed samples are remarkably similar: the potentials at which the maximum intensity for each redox species is observed are consistent throughout. This result confirms the integrity of the dynamic behaviour around the active site of Hyd1 in single crystals, thus showing that observations made in the crystalline state of Hyd1 are mechanistically relevant to the enzyme in solution, and to PFE studies of enzyme activity.

### Potential-controlled single-crystal redox titration of Hyd1, pH 8.0, monitored by IR microspectroscopic electrochemistry

**Fig. 4:**
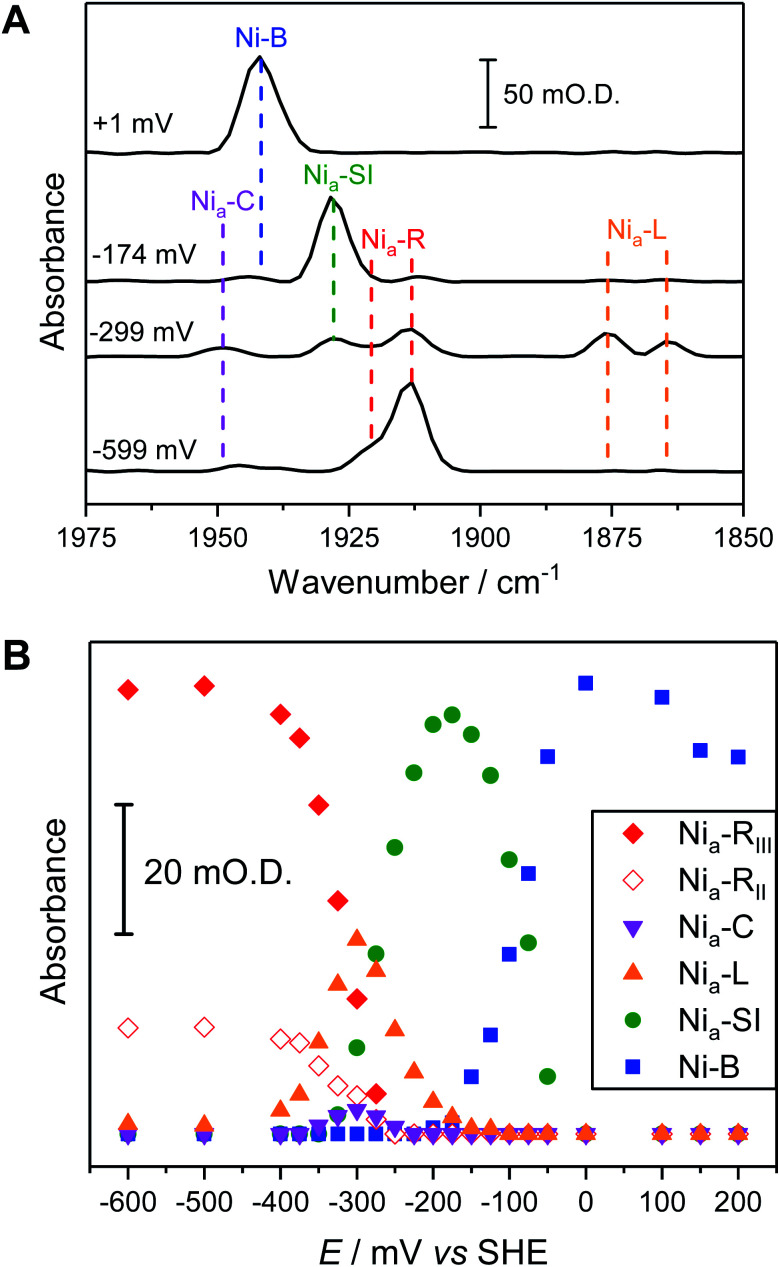
Electrochemical redox titration of a Hyd1 crystal at pH 8.0 recorded using the IR microspectroscopic electrochemical technique. (A) Baseline-corrected IR spectra showing the *ν*_CO_ region at selected potentials. Dotted lines show the wavenumber positions of the intrinsic *ν*_CO_ bands of the active site redox states of Hyd1. Wavenumber positions are given in Table S3.† For IR spectra of the *ν*_CN_ and *ν*_CO_ regions across the full potential range of −600 to +200 mV see Fig. S10.† (B) The speciation curves illustrate how the absorbance of the *ν*_CO_ peaks of Hyd1 active site species vary with potential at pH 8.0.

Hydrogenases catalyse H_2_ activation *via* a series of exquisitely-timed electron transfer and proton-coupled electron transfer steps (Scheme S1†). Studies of hydrogenase activity and spectroscopic properties have been reported at a range of pH in order to establish details of the proton inventory during catalysis.^35,36,69^ Crystals of Hyd1 are stable over a relatively wide pH range,^32^ and by pre-soaking crystals in pH-adjusted crystal stabilisation buffer prior to loading into the microspectroscopic-electrochemical cell we can carry out single crystal redox titrations at different pH values. Here, we focus particularly on probing the known pH-dependence of Ni_a_–C and Ni_a_–L tautomerism^23^ in the crystalline state. Fig. 4A presents IR spectra in the *ν*_CO_ region, extracted from an oxidative redox titration of a single Hyd1 crystal measured at pH 8.0. The representative IR spectra in Fig. 4A were chosen to coincide with the maximum intensity for each redox species from the full potential titration shown in Fig. 4B (spectra including both *ν*_CN_ and *ν*_CO_ regions, raw data, and representative fitted spectra, are presented in Fig. S10–S12†). Once again, all active site redox species are detected in specific potential windows of the redox titration, including the Ni_a_–R and Ni_a_–L sub-states. An additional species with *ν*_CO_ at 1938 cm^−1^ is evident at potentials above +100 mV, and accounts for the apparent loss of Ni–B (1942 cm^−1^) at these potentials in Fig. 4B. The potential dependence of this 1938 cm^−1^ species (Fig. S13†) is similar to the potential of the [Fe_4_S_3_]^5+/4+^ proximal cluster transition^39,70^ and could be related to formation of the superoxidised proximal cluster (Table S4†). Further investigation of this behaviour is a target for future studies.

### pH-dependent behaviour of the active site and surroundings

**Fig. 5:**
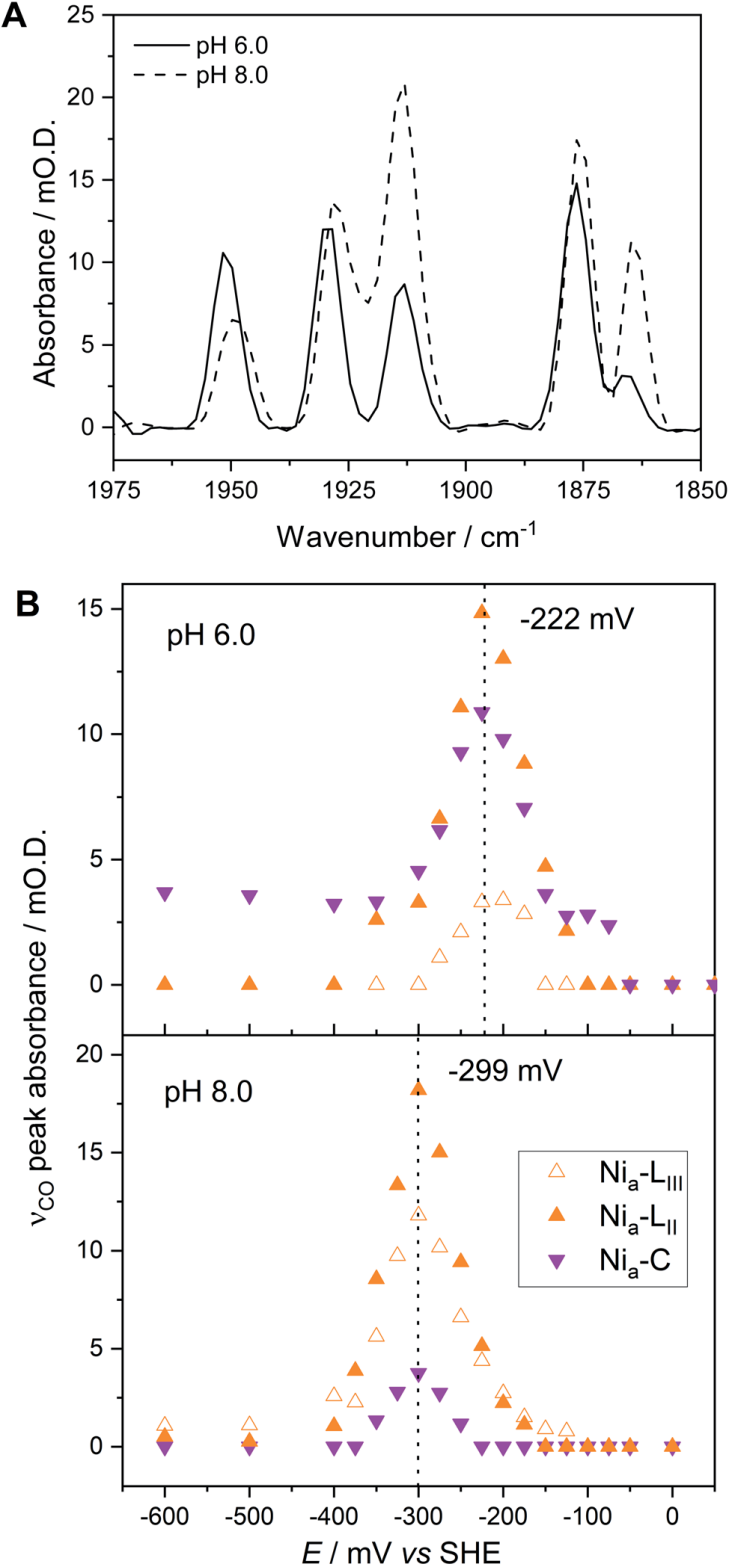
The pH dependence of the Ni_a_–C and Ni_a_–L redox species. (A) Baseline-corrected IR spectra showing the *ν*_CO_ region of Hyd1 crystals, recorded at pH 8.0 (−299 mV) and pH 5.9 (−222 mV). (B) Speciation as a function of potential as measured by IR microspectroscopic-electrochemistry. The potential at which maximum *ν*_CO_ peak intensity for each species is observed is marked.

Fig. 5A compares IR spectra in the *ν*_CO_ region at both pH 5.9 and pH 8.0, extracted from redox titrations at −222 mV (pH 5.9, Fig. 3A) and −299 mV (pH 8.0, Fig. 4), potentials at which the intensities of the Ni_a_–C and Ni_a_–L states are maximal. We observe three main differences in both the titration data and spectra at pH 5.9 in comparison to pH 8.0:

(1) The relative populations of Ni_a_–C and total Ni_a_–L, and of the individual Ni_a_–L_II/III_ sub-states, are pH dependent.

(2) The equilibrium midpoint potential for transitions between each redox level shifts to more negative potentials at pH 8.0.

(3) The *ν*_CO_ peak positions for all redox states shift to lower wavenumbers at pH 8.0 relative to pH 5.9 (Fig. 5A and Table S3†).

The redox titration data reported in Fig. 3 (at pH 5.9) and Fig. 4 (at pH 8.0) explicitly show contributions from two Ni_a_–R sub-states present in Hyd1, Ni_a_–R_II_ and Ni_a_–R_III_. The peak positions at each pH are provided in Table S3.† For simplicity in Fig. 3 and 4 we combined contributions from two Ni_a_–L sub-states. Fig. 5B replots the titration data, highlighting the Ni_a_–C/Ni_a_–L potential window and showing individual contributions for Ni_a_–C and both Ni_a_–L states observed in Hyd1, Ni_a_–L_II_ (*ν*_CO_ at 1877 cm^−1^ at pH 5.9) and Ni_a_–L_III_ (*ν*_CO_ at 1866 cm^−1^ at pH 5.9). We have previously demonstrated a pH-dependent tautomeric equilibrium between the Ni_a_–C and Ni_a_–L species in Hyd1 (Scheme 1).^23^ Here we observe the same shift in equilibrium towards Ni_a_–L at higher pH (Fig. 5B), showing that Ni_a_–C/Ni_a_–L tautomerism is maintained in the crystalline state. This observation is critical, as it suggests that crystallisation does not perturb proton transfer equilibria in the vicinity of the [NiFe] active site, in addition to the unperturbed electron transfer redox equilibria demonstrated by the equilibrium redox titrations in Fig. 3 and 4.

In addition to providing evidence of Ni_a_–C/Ni_a_–L tautomerisation in the crystalline state, it is clear from Fig. 5 that the relative proportions of each Ni_a_–L sub-state also vary with pH, consistent with the behaviour of electrode-adsorbed Hyd1 (Fig. S14†) where the population of Ni_a_–L_II_ remains roughly constant above pH 6.^71,72^ The mechanistic role of the Ni_a_–L sub-states as sequential intermediates in proton transfer to/from the [NiFe] active site has been demonstrated in photo-triggered potential jump measurements on soluble hydrogenase 1 (SH1) from *P. furiosus*,^36^ and cryogenic photolysis of the [NiFe] hydrogenase from *D. vulgaris* Miyazaki F.^33,34^ The most common representation of ‘Ni_a_–L’ invokes protonation of a terminal cysteine–S ligand to Ni at the active site. Evidence of cysteine–S protonation in the Ni_a_–L_I_ sub-state has been reported in the *D. vulgaris* Miyazaki F [NiFe] hydrogenase, where H/D labelling suggested the presence of an S–H stretching vibration in Ni_a_–L_I_.^37^ We have previously noted that the Ni_a_–L_I_ sub-state does not accumulate significantly, if at all, in O_2_-tolerant [NiFe] hydrogenases such as Hyd1,^22^ and this behaviour is maintained in the crystalline state. Computational modelling studies of the active site suggest that deprotonation of a terminal cysteine thiol ligand to Ni causes *ν*_CO_ to shift to lower energy by *ca.* 30 cm^−1^.^73^ This shift upon deprotonation matches the difference in *ν*_CO_ observed between Ni_a_–L_I_ and Ni_a_–L_II/III_ for a range of [NiFe] hydrogenases,^22,72^ leading us to postulate that a terminal cysteine thiol is not present in either of the Ni_a_–L_II_ or Ni_a_–L_III_ sub-states. The high Hyd1 concentration (8 mM) within single crystals allows us to test this hypothesis further through direct observation of the S–H stretching region (*ca.* 2450–2600 cm^−1^). Difference spectra are particularly sensitive to changes in cysteine–S protonation between individual redox states.^37^ Potential-induced single crystal difference spectra (Fig. S15,† calculated from raw in crystallo microspectroscopy data) suggest that there is no change in cysteine–S protonation between the Ni_a_–R_II/III_, Ni_a_–C, Ni_a_–L_II/III_, and Ni_a_–SI redox states in Hyd1. Therefore we find no evidence of S–H bond formation in the Ni_a_–L_II_ or Ni_a_–L_III_, and Ni_a_–R_II_ or Ni_a_–R_III_ sub-states of Hyd1 (Fig. S15†). Whilst the apparent lack of an S–H resonance in crystalline Hyd1 does not conclusively rule out cysteine thiol formation in Ni_a_–L_II/III_ or Ni_a_–R_II/III_, the high S/N and intensity of spectra recorded from concentrated, crystalline Hyd1 would provide the ideal scenario for detecting any low-intensity S–H resonances.

It is generally accepted that a glutamate residue (E28 in Hyd1 numbering) close to the active site is critical for proton transfer during Ni_a_–L formation from Ni_a_–C,^32,36,37,74^ and the primary proton acceptor during this transition in *P. furiosus* SH1 has been shown to have a p*K*_a_ of approximately 7.^35^ It is therefore possible that deprotonation of E28 is required for enrichment of Ni_a_–L_III_ at pH 8.0. However the Ni_a_–L_II_ sub-state has a considerably lower apparent p*K*_a_ ∼ 5 (Fig. S14†), implying that deprotonation of E28 is not required for Ni_a_–L_II_ formation from Ni_a_–C in *E. coli* Hyd1.

The spectra of Hyd1 in Fig. 5A show an apparent peak shift and broadening of the Ni_a_–C *ν*_CO_ band upon change of pH. Peak fitting of these data (Table S3†) suggests that the Ni_a_–C peak actually contains contributions from two distinct *ν*_CO_ resonances for Ni_a_–C, at 1951 cm^−1^ and 1947 cm^−1^, with the lower wavenumber species enriched at pH 8.0. This is consistent with the observations of Greene *et al.*, who noted a pH equilibrium between two forms of Ni_a_–C in *P. furiosus* SH1 with an apparent p*K*_a_ of 6.8.^36^ The mechanistic relevance of this is not clear, although Greene *et al.* have postulated that protonation/deprotonation of glutamate E28 could account for the pH-dependent shift in the *ν*_CO_ position of Ni_a_–C.^36^ In Fig. 5A we also observe a pH-dependent shift in the *ν*_CO_ band of Ni_a_–SI (Table S3†).

### The transition from Ni_a_–C to Ni_a_–SI

**Fig. 6:**
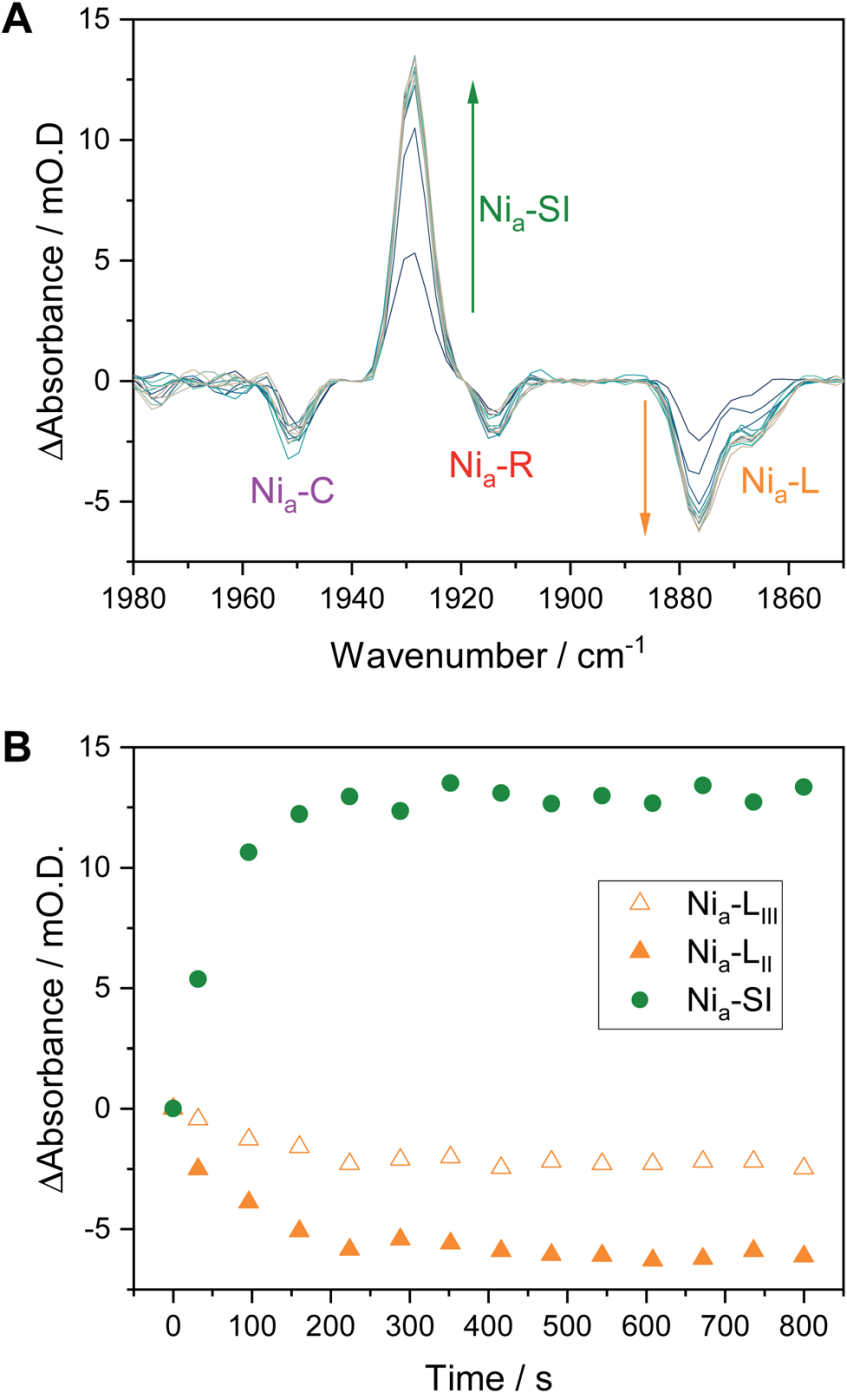
Ni_a_–L to Ni_a_–SI transition in Hyd1 pH 5.9 crystals following a potential step from −197 mV to −172 mV *vs.* SHE. (A) Difference spectra following the potential step showing the increase in Ni_a_–SI and decrease in Ni_a_–L species over time. Any change in Ni_a_–R and Ni_a_–C is not apparent after the initial IR spectrum. (B) Time dependence of the change in Ni_a_–SI and Ni_a_–L species. Traces including other active site redox species are in the ESI, Fig. S18.†

We have previously shown that redox transitions involving chemical steps such as proton transfer appear to be retarded in the crystalline state.^14^ By continuously recording IR spectra during equilibration after each potential step in an electrochemical redox titration, we can monitor these kinetic aspects of equilibration in the crystals. Fig. 6A shows a series of difference spectra of a Hyd1 crystal at pH 5.9, following equilibration after a small oxidative potential step from −197 mV to −172 mV, *i.e.* a positive potential step from where Ni_a_–C and the Ni_a_–L states are maximal (Fig. 3A). The difference spectra are presented as −172 mV *minus* −197 mV, and the raw experimental data and baseline corrected spectra are shown in Fig. S16 and S17.† The corresponding change in absorbance of the Ni_a_–SI and Ni_a_–L *ν*_CO_ resonances as a function of time is plotted in Fig. 6B and reveals an interesting relationship between the Ni_a_–SI and Ni_a_–L species. The first spectrum recorded after the potential step shows that all redox species present experience a step change in their relative intensities, implying equilibration of the applied potential throughout the crystal during the first 32 s after the potential step (see Fig. S18† for a plot including the Ni_a_–C and Ni_a_–R states). Subsequent spectra during the equilibration process show further slow interchange between Ni_a_–L and Ni_a_–SI, whilst Ni_a_–C and Ni_a_–R absorbances remain approximately constant after the first spectrum.

Previous studies have shown the involvement of the Ni_a_–L sub-states as an on-pathway intermediate between Ni_a_–C and Ni_a_–SI during catalysis.^23,33,36^ Fast kinetic methods, capable of probing redox chemistry of the [NiFe] active site with sub-turnover frequency time resolution, were necessary to conclusively confirm the catalytic competence of the Ni_a_–L states. Here we are able to access similar information without the need for fast time resolution. The electrochemical control afforded by the microspectroscopic-electrochemical cell, in combination with solution redox mediators, provides a source or sink of electrons that are available to the crystalline protein on a timescale that is clearly faster than some chemical steps in crystallo. This is evident due to the relatively fast equilibration of the Ni_a_–C and Ni_a_–R states (which differ only in active site redox state rather than protonation in Hyd1, Scheme 1), relative to Ni_a_–L and Ni_a_–SI (which additionally require a proton transfer step) in Fig. 6. We have previously noted that O_2_-tolerant [NiFe] hydrogenases do not accumulate either the Ni_a_–R_I_ or Ni_a_–L_I_ sub-states, and instead favour the Ni_a_–R_II/III_ and Ni_a_–L_II/III_ sub-states.^22^ We find no evidence for cysteine–S protonation in the Ni_a_–R_II/III_ and Ni_a_–L_II/III_ sub-states in crystallo, and the faster rate of the Ni_a_–R → Ni_a_–C transition implied by the data in Fig. 6 and S17 is consistent with this transition involving only electron transfer in the case of Hyd1. The fact that the Ni_a_–L to Ni_a_–SI transition is apparently rate limiting during this potential step is consistent with involvement of both proton and electron transfer.

We have previously discussed possible mechanistic implications of the unusual high-potential [4Fe3S]^5+/4+/3+^ cluster proximal to the active site found in O_2_-tolerant [NiFe] hydrogenases, in particular concerning whether proton-coupled electron transfer between Ni_a_–L and Ni_a_–SI occurs *via* a concerted or stepwise mechanism.^22^ From the work of the groups of Hirota and Dyer it is known that onwards formation of Ni_a_–SI from Ni_a_–C, *via* Ni_a_–L, requires the proximal iron-sulfur cluster to be capable of receiving an electron, *i.e.* to be in an oxidised state.^33,68^ The potential of both the [4Fe3S]^4+/3+^ and [4Fe3S]^5+/4+^ transitions of the proximal cluster in Hyd1 are relatively high, +3 mV and +230 mV respectively at pH 6 (see Table S4†),^38,39,70^ and so the proximal cluster of Hyd1 will largely be in the [4Fe3S]^3+^ state at equilibrium at the potentials applied in Fig. 6. In the O_2_-sensitive hydrogenases the proximal cluster is a standard cubane [4Fe4S]^2+/+^ cluster and its potential is closer to the potential of the H^+^/H_2_ couple at neutral pH.^38^ The electron transfer necessary for the Ni_a_–R → Ni_a_–C and Ni_a_–C → Ni_a_–SI transitions is therefore hindered in Hyd1 relative to O_2_-sensitive [NiFe] hydrogenases, and we suggest that this may be responsible for the fact that the Ni_a_–R_I_ and Ni_a_–L_I_ sub-states do not accumulate in Hyd1. In combination with our earlier hypothesis that cysteine–S protonation is not present in either of the Ni_a_–L_II_ and Ni_a_–L_III_ sub-states of Hyd1, we tentatively suggest two possible proton-coupled electron transfer mechanisms for the conversion between Ni_a_–L and Ni_a_–SI. In the first mechanism, concerted proton and electron transfer occurs during Ni_a_–SI formation as previously reported by Dyer and co-workers.^35^ In the second mechanism, likely prevalent in O_2_-tolerant hydrogenases such as Hyd1, proton and electron transfer is stepwise due to electron transfer between the active site and proximal cluster becoming limiting in the presence of the unusual high potential [4Fe3S] cluster. Proton transfer is relatively unaffected, as the active site structure and surrounding amino acids are highly conserved, and so H^+^ can leave the active site ahead of electron transfer during the Ni_a_–L → Ni_a_–SI transition.

### Scope for crystal structures of well-defined states

Prolonged exposure to the mediator cocktail and application of potential have no effect on the ability of Hyd1 crystals to diffract X-rays (Fig. S19 and Table S5†), suggesting electrochemical manipulation of Hyd1 crystals offers the exciting prospect of producing molecular models for intermediates of catalysis that have so far been inaccessible to structure determination. The exquisite control of electrochemical potential afforded by the electrode allows crystals to be precisely poised under conditions that favour formation of only the intermediate of interest, for example the most reducing potentials applied allowed accumulation of pure Ni_a_–R. This advantage contrasts strongly with the reduction of [NiFe] hydrogenase crystals by H_2_ which generates complex mixtures of states that are less suitable for structure determination.^10,25^ Manipulation of pH offers a further dimension to the control over speciation of the crystalline enzyme. Such control offers a more rational approach to obtaining structures for catalytic intermediates than has previously been possible and eliminates the need for low activity variants,^75^ inhibitors,^76^ or transition-state analogues^77^ that have been mainstays of classical (pre-XFEL) time-resolved structure determination from single crystals. Furthermore, our technique offers the possibility of finally linking the spectral fingerprints of each intermediate to a defined configuration of the active site and spectral changes occurring during turnover with specific atomic motions.

## Conclusions

Working with single crystals of Hyd1, we have described a method that allows complete control over the redox state of crystalline proteins. The measurements reported confirm that protein crystals can be viewed as a dynamic system where all known states and intermediates are reliably accessible. High protein concentrations in the crystalline state allow us to record spectra with high signal/noise ratios, facilitating assignment of the *ν*_CO_ bands of each active site state. The active site *ν*_CO_ and *ν*_CN_ band positions and redox chemistry in single crystals of Hyd1 are consistent with previously reported behaviour, providing compelling evidence that crystallisation does not change the immediate environment or chemical properties of the active site relative to solution-phase protein. All known states of the Hyd1 active site can be generated under fine potential control, and detailed redox titrations recorded from single crystals match those for Hyd1 in solution or adsorbed on an electrode. These single crystal measurements thus bridge the gap between structural, spectroscopic, and activity-based biophysical methods, and provide confirmation that the behaviour of proteins in a range of physical states are comparable. The pH of the crystals is also readily manipulated, and all aspects of proton transfer, including Ni_a_–C/Ni_a_–L tautomerism are retained in the crystalline state. Detailed electrochemically-controlled redox titrations of the Hyd1 active site demonstrate the importance of single crystal microspectroscopy as a complementary method to protein crystallography, and could be used for spectroscopic characterisation post-X-ray diffraction to provide confirmation of the redox state solved. This aspect is particularly important given the often complex mixtures of redox states that are present over wide potential windows. Likewise, the ability to generate pure redox states across a narrow potential window, as demonstrated here, allows infrared microspectroscopic-electrochemical methods to deliver a roadmap for how to enrich and prepare individual redox states in crystallo for downstream structure determination. Crystal structures can thus be directly relevant to redox states probed during the catalytic cycle. In addition to electrochemical navigation of the redox states, through careful control over pH it is possible to access different sub-states (*e.g.* of Ni_a_–R and Ni_a_–L), providing an extra dimension of control for future crystallographic studies.

Of further significance, through electrochemical control over single crystals we are able to access retarded reaction steps that are otherwise hidden in steady-state catalytic studies. The use of both positive and negative potential steps reveals details of proton-coupled electron transfer to and from the active site, and has allowed us to hypothesise sequential, rather than concerted, proton and electron transfer during the Ni_a_–L → Ni_a_–SI transition in O_2_-tolerant [NiFe] hydrogenases such as Hyd1. This is in contrast to concerted proton and electron transfer observed in O_2_-sensitive [NiFe] hydrogenases.

The method reported here, already extended to crystals of *C. pasteurianum* [FeFe] hydrogenase I,^78^ is likely to have general relevance in structure–function studies of complex redox metalloenzymes.

## Data availability

Data in support of this article are included as part of the ESI.†

## Author contributions

P. A. A. and K. A. V. were responsible for conceptualisation of the study. Investigation was carried out by P. A. A., S. E. T. K.-P., R. M. E., S. B. C., A. B., S. M., J. S. R., R. H., A. J. H., G. C., M. D. F., and K. A. V. Methodology and analysis were carried out by P. A. A., S. E. T. K.-P., R. M. E., G. C., and M. D. F. All authors were involved in writing and visualisation of the manuscript.

## Conflicts of interest

There are no conflicts to declare.

## Supplementary Material

SC-012-D1SC01734A-s001
