# Supplementary material for: The crystalline state as a dynamic system: IR microspectroscopy under electrochemical control for a [NiFe] hydrogenase
Source: Chem Sci. 2021 Jun 3;12(39):12959–70. doi: 10.1039/d1sc01734a (PMC8514002; doi:10.1039/d1sc01734a)
Supplement: SC-012-D1SC01734A-s001 [file SC-012-D1SC01734A-s001.pdf]

Electronic Supplementary Material (ESI) for Chemical Science.
This journal is © The Royal Society of Chemistry 2021

The crystalline state as a dynamic system: IR microspectroscopy under electrochemical control for a [NiFe] hydrogenase

Philip A. Ash,[a,b,c*] Sophie E. T. Kendall-Price,[a] Rhiannon M. Evans,[a] Stephen B. Carr,[a,d] Amelia Brasnett,[a] Simone Morra,[a,e] Jack S. Rowbotham,[a] Ricardo Hidalgo,[a,+] Adam J. Healy,[a] Gianfelice Cinque,[f] Mark D. Frogley,[f] Fraser A. Armstrong,[a] Kylie A. Vincent[a*]

Supporting Information

Table of Contents

Supplementary Methods 3

Single-crystal IR microspectroscopic-electrochemical experiments 3

Figure S1. Schematic representation of the IR microspectroscopic-electrochemical cell 3

Solution infrared spectroscopic-electrochemical measurements 4

Infrared spectroscopic-electrochemical measurements of electrode-adsorbed Hyd1 4

Calculation for number of active sites illuminated with IR microspectroscopy per unit of crystal volume 6

Scheme S1. The catalytic cycle of Hyd1, including oxidised inactive state Ni-B 7

Table S1. Mid-point potentials (E_m) and peak-to-peak separations (ΔEp) of redox mediators 8

Figure S2. Cyclic voltammogram demonstrating electrochemical control within the infrared microspectroscopic-electrochemical cell 9

Figure S3. Illustration of the solvent channels within a crystal of Hyd1 10

Figure S4. Raw IR spectrum of a single Hyd1 crystal at pH 5.9 11

Figure S5. Raw IR spectrum of a single Hyd1 crystal at pH 5.9 poised at −597 mV 12

Figure S6. Electrochemical oxidative titration of a single crystal of Hyd1 at pH 5.9. 13

Figure S7. Raw IR spectra for pH 5.9 oxidative titration 14

Figure S8. Chronoamperometry 15

Figure S9. Representative potential-dependent spectra at pH 5.9 16

Table S2. Comparing active site species at pH 5.9 17

Figure S10. Electrochemical oxidative titration of a single crystal of Hyd1 at pH 8.0. 18

Figure S11. Raw IR spectra for pH 8.0 oxidative titration 19

Figure S12. Representative potential-dependent spectra at pH 8.0 20

Figure S13. A comparison of pH 5.9 and pH 8.0 21

Table S3. The pH dependence of active site ν_{CO} 22

Table S4. Proximal cluster potentials 23

Figure S14. The relative populations of Ni_a-C, Ni_a-L_{II}, and Ni_a-L_{III} are pH dependent 24

Figure S15. Single crystal difference spectra show no evidence of cysteine-S protonation in either Ni_a-$R_{II/III}$ or Ni_a-$L_{II/III}$ 25

Figure S16. The transition of Ni_a-L to Ni_a-SI in single crystals of Hyd1 at pH 5.9 26

Figure S17. The transition of Ni_a-L to Ni_a-SI in a single crystal of Hyd1 at pH 5.9 27

Figure S18. Time dependence of the active site redox species in a single crystal of Hyd1 at pH 5.9...28

Figure S19. X-ray diffraction data for mediator soaked Hyd1 crystals 29

Table S5. X-ray data collection statistics 30

Supplementary References 32

Supplementary Methods

Single-crystal IR microspectroscopic-electrochemical experiments

Electrochemical cell design

Figure S1 shows a schematic representation of the electrochemical cell used for microspectroscopic-electrochemical measurements, based around previously-reported electrochemical cells.[1–4] A glassy carbon rod working electrode (4 mm diameter, Alfa Aesar) was sealed into a brass connector using silver-loaded epoxy resin (RS Components). This working electrode assembly was then sealed, using epoxy resin (Araldite), into a custom-built cell 'puck' constructed from either PEEK or Delrin. A graphite ring counter electrode, depth approximately 5 mm, was cut from a graphite tube (Goodfellow, 1.6 mm wall thickness) and was sealed into a groove in the cell puck using epoxy resin. Connection to the counter electrode was achieved using a Pt wire (Surepure Chemetals, 99.99 %, 26 gauge) fixed to the rear of the counter electrode (before sealing into the cell) using silver-loaded epoxy resin. The cell surface, glassy carbon and graphite ring electrodes were polished to a mirror finish using successive grades of silicon carbide paper (to 4000 grit, Kemet). The polished cell was then washed by ultrasonication in ultra-high purity water. A miniature Ag/AgCl reference electrode (3 M KCl, eDAQ, 2 mm diameter) was positioned close to the electrodes to minimize uncompensated resistance. The reference electrode was removed prior to polishing, and sealed into the cell during measurements using silicone sealant (Dowsil, SE 9187L Silicone RTV).

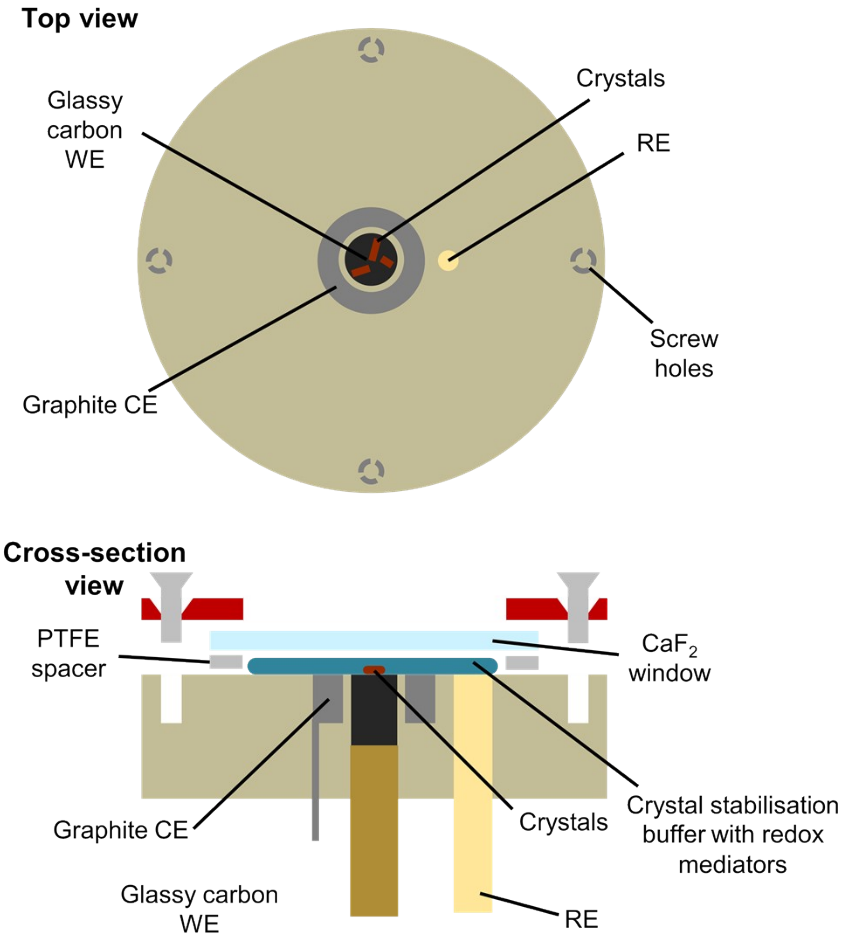

Figure S1. Schematic representation of the IR microspectroscopic-electrochemical cell used in this work. The arrangement of the working, reference and counter electrodes (WE, RE, CE, respectively) are shown. Not to scale.

IR microspectroscopic data collection

Single crystal spectra were recorded using 15 × 15 μm^2 knife-edge apertures in the detection beampath to define the volume of crystal illuminated during a measurement. Spectra were recorded relative to a background spectrum of the crystal stabilisation buffer and redox mediator mixture, recorded in situ prior to each electrochemical redox titration using a reflection from the bare electrode surface approximately 30-45 μm from the crystal under investigation. We found that this provides a reasonable method to minimise interference artefacts due to roughness of the electrode surface and high brightness of the synchrotron IR source. We have previously shown that crystals of Hyd1 are stable over several days in the electrochemical cell, with no detectable dissolution of protein. All raw data presented in this work therefore represent absolute spectra of the crystal under investigation, and baseline-corrected data are free from significant artefacts in the background spectrum.

Solution infrared spectroscopic-electrochemical measurements

Solution spectroscopic-electrochemical measurements were carried out in a similar manner to those previously reprted.[5,6] A phosphate-buffered Nafion solution was prepared by titrating a 1:1 v/v mixture of Nafion 117 (Sigma Aldrich, 10% aqueous dispersion) and K_2HPO_4 (BDH, 100 mM) to pH 6.0 using small quantities of concentrated NaOH (BDH). A 2.5 μL aliquot of phosphate-buffered Nafion was then mixed with 2.5 μL of carbon black particle dispersion (XC72R, DuPont, 20 mg mL^{-1}) and 20 μL Hyd1 solution (*ca* 10 mg mL^{-1}), and allowed to concentrate by evaporation to a volume of *ca* 15 μL. The Nafion-carbon-Hyd1 mixture was deposited onto the surface of a silicon prism (Crystal GmBH) mounted on an ATR-IR accessory (GladiatIR, Pike Technologies) in an anaerobic, dry N_2-filled glovebox (Glove Box Technology, <1 ppm O_2, <−75 °C dew point). The mixture was allowed to concentrate under N_2 to a final volume of *ca* 3-5 μL, at which point the carbon black particle dispersion forms a 3D particle network electrode encasing concentrated Hyd1 in the aqueous Nafion. Lateral contact was achieved using carbon paper (AvCarb P50, Ballard Power Systems), and electrical contact to the composite working electrode was made using a carbon rod (WH Smith, 0.9 mm HB) sealed into the body of an electrochemical cell.[6,7] The electrochemical cell contained additional feedthroughs for a fritted counter electrode (Pt wire) and a homemade miniature saturated calomel reference electrode (SCE) as previously described.[7] The ATR-IR solution spectroscopic-electrochemical cell was filled with N_2-saturated, phosphate-buffered electrolyte solution (50 mM, pH 6, 100 mM KCl). A closed loop of N_2-purged electrolyte was pumped through the cell during data collection to prevent build-up of any trace H_2 produced by Hyd1.

IR spectra were recorded at a range of applied potentials, allowing a minimum of 15 minutes equilibration time after each potential step. Equilibration was confirmed by the absence of changes to the ν_{CO} and ν_{CN} bands of the active site over five successive spectra. Spectra were recorded using an Agilent 680-IR spectrometer controlled by ResPro 4 software, as an average of 1024 interferograms (*ca* 360 s measurement time). Electrochemical control was provided by an Autolab 128N potentiostat (Metrohm), and potentials (*E*) are reported relative to the standard hydrogen electrode (SHE) using the conversion E(mV *vs* SHE) = E(mV *vs* SCE) + 241 mV at 25 °C.[8] Baseline correction, and all subsequent data analysis was carried out using OriginPro software (OriginLab Corp., version 9.1).

Infrared spectroscopic-electrochemical measurements of electrode-adsorbed Hyd1

The data collected from electrode-adsorbed Hyd1 reported in this manuscript were reproduced from data previously reported by us in Hidalgo *et al.*.[7] Simultaneous IR spectroscopic-electrochemical measurements were carried out using the ATR-IR cell and protein film IR electrochemistry (PFIRE) method. Briefly, a 50 μL aliquot of as-isolated Hyd1 (6.3 mg mL^{-1}) was exchanged into potassium phosphate buffer (15 mM, pH 5.8) by dilution and re-concentration using a 50 kDa molecular weight cut-off microcentrifugal concentrator (Amicon Ultra 0.5 mL, Merck). The enzyme sample was then

mixed with 5 µL of a carbon black particle suspension (Black Pearls 2000, Cabot Corporation, dispersed in water by sonication to a loading of 20 mg mL^{-1}) and left at 0 °C for 2 h to allow for enzyme adsorption. The particles modified with Hyd1 were then washed with buffer to remove un-adsorbed enzyme before re-concentration to a carbon black loading of 20 mg mL^{-1}. Enzyme adsorption was carried out in a N_2-filled glovebox (< 1 ppm O_2, Glove Box Technology Ltd.).

An aliquot (ca 1-2 µL) of Hyd1-modified particles was deposited onto the surface of a Si internal reflection element (Crystal GmBH) mounted onto an ATR-IR accessory (GladiatIR, PIKE technologies). Lateral electrical connection was provided by carbon paper (Toray, TGP-H-030) placed on top of the deposited particle film. A spectroscopic-electrochemical cell body housing a miniature saturated calomel reference electrode, Pt wire counter electrode, carbon rod working electrode connection, and an inlet and outlet to allow solution flow, was sealed onto the ATR-IR accessory and filled with Ar-saturated buffer. Ar-saturated buffer was flowed through the cell throughout measurements to prevent build-up of any trace H_2 produced. A mixed buffer solution (pH 6.0, MES, HEPES, TAPS, CHES, NaOAc, 15 mM each) containing NaCl (100 mM) as supporting electrolyte was used as the flow solution. The aerobically-purified Hyd1 was reductively activated under 1 bar H_2 at −594 mV for at least 1 hour before collecting redox titration data.

IR spectroscopic data were collected using an Agilent 680-IR spectrometer controlled using ResPro 4.0 software. Baseline correction and all subsequent data handling was carried out using OriginPro software (OriginLab Corp., version 9.1).

Calculation for number of active sites illuminated with IR microspectroscopy per unit of crystal volume
Approximate Unit cell volume = 93x97×183 Å^3 = 1.642×10^6 Å^3

Unit cells / µm^3 = 609,013

In Spacegroup $P2_12_12_1$ the unit cell contains 4 asymmetric units

Thus Hyd1 molecules / µm^3 = 2,436,000

Protein is a dimer, therefore 4.87×10^6 active sites / µm^3

Typically, a sampling area of 15×15 µm^2 is used in the IR microspectroscopy experiments:

Active sites sampled during experiment = 1.1×10^9 for each µm of crystal depth.

Conversion of number of active sites to effective molarity of active sites
Molar concentration of active site = (No. active sites/Avogadro's constant) / Volume in dm^3

$$\frac{4.87 \times 10^{6} \div 6.022 \times 10^{23}}{1 \times 10^{-15}} = \sim 8\ mM$$

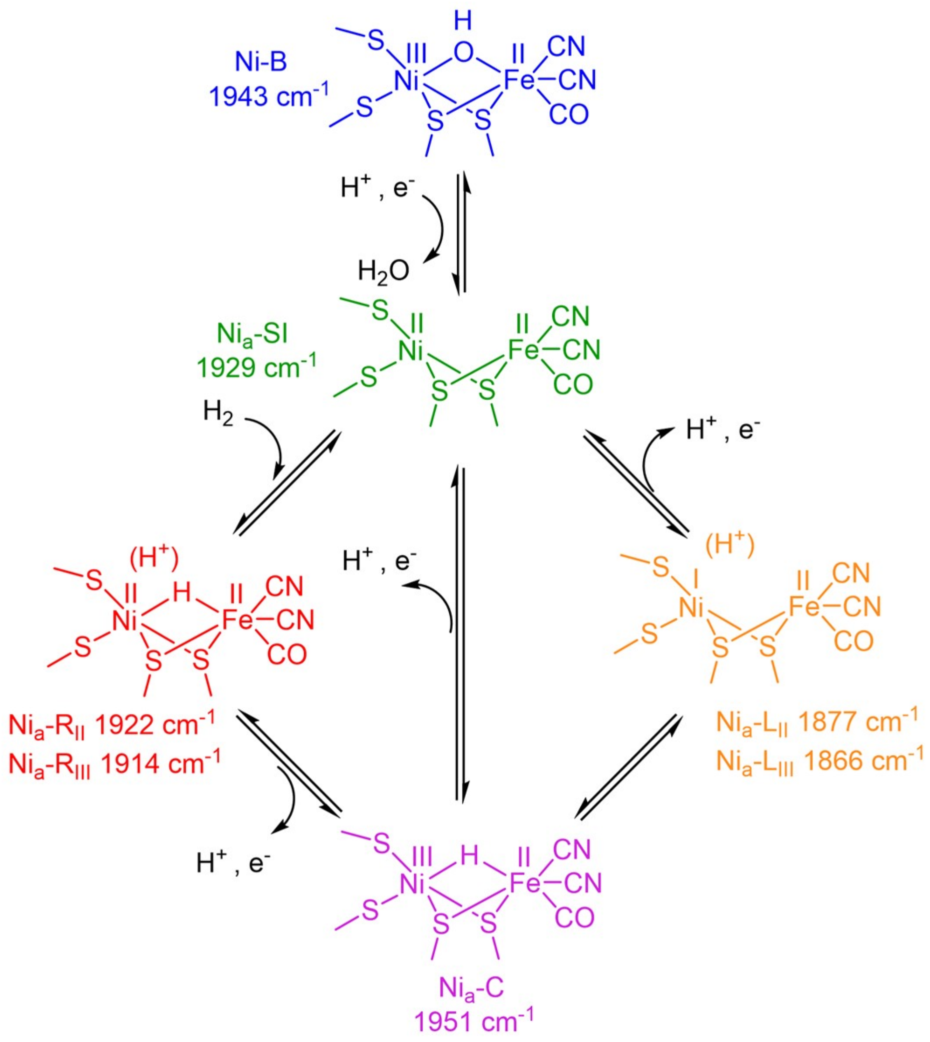

Scheme S1. The catalytic cycle of Hyd1, including oxidised inactive state Ni-B. States are coloured as in Scheme 1 and denoted in spectra throughout. Viewed in the direction of H_2 oxidation, H_2 is thought to bind at the level of Ni_a-SI to form a transient Michaelis complex. The H_2 molecule is polarised between an active site metal (Lewis acid) and a nearby base, the identity of which is still under debate. Candidates include a terminal cysteine thiol (C576 in Hyd1 numbering) in the primary coordination sphere of the active site Ni, or the pendant arginine of R509 located ca 4 Å in a "canopy" above the bridging position between Ni and Fe.[9–13] Abstraction of the proton from H_2 leaves a hydride in a bridging position between the two metals, forming the most reduced state of the active site generally termed Ni_a-R. Next, the Ni is oxidised from Ni^{II} to Ni^{III}, the electron being transferred to the proximal FeS cluster, and the proton abstracted from H_2 is transported away from the active site forming Ni_a-C. From here a proton and an electron must be lost from the active site to re-form Ni_a-SI. In the case of Hyd1 the oxidation of Ni_a-C to Ni_a-SI has been shown to occur via Ni_a-L: the bridging hydride moves as a proton, via C576 to E28,[14,15] leaving its two electrons on the Ni, forming Ni^{I}. Oxidation of Ni^{I} to Ni^{II} occurs via transfer of the second electron to the proximal cluster. However, this electron transfer step is likely retarded by the high potential of the FeS clusters of Hyd1,[16] ultimately leading to the ability to be able to identify Ni_a-L as a true intermediate in the catalytic cycle.[7,17,18] The Ni-B inactive state can be formed by the oxidation of the active site, either by application of high potentials, or by the binding of O_2 (which requires 3 protons and 3 electrons to form Ni-B, 1 electron reduction of which re-forms Ni_a-SI).

Table S1. Mid-point potentials (E_m) and peak-to-peak separations (ΔEp) of redox mediators used, measured in the infrared microspectroscopic-electrochemical cell described in Figure S1 at pH 5.9. Literature values of E_m are provided for comparison.

Redox mediator	Molecular structure	Literature E_m / mV[(a)]	Measured E_m / mV[(b)]	ΔE_p / mV
2,6-dichloroindophenol sodium salt (DCPIP)	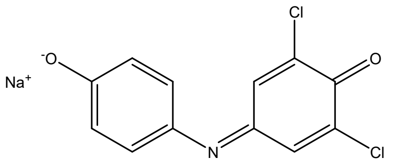	+217[19,20]	+317	46
Phenazine methosulfate	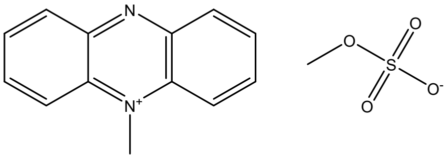	+80[20]	+139	56
Indigo carmine	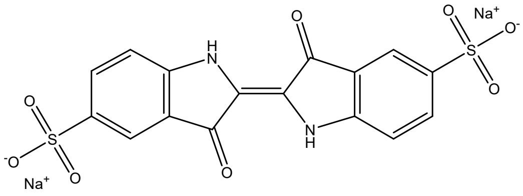	-125[21]	-29	71
Anthraquinone-2-sulfonate	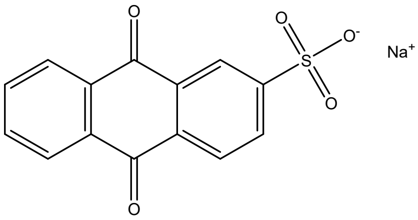	-225[22]	-151	85
Methyl viologen	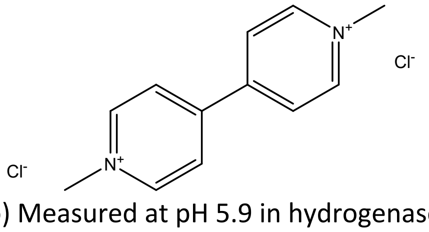	-446[23–25]	-414	44

(a) Reported at pH 7. (b) Measured at pH 5.9 in hydrogenase crystal stabilisation buffer.

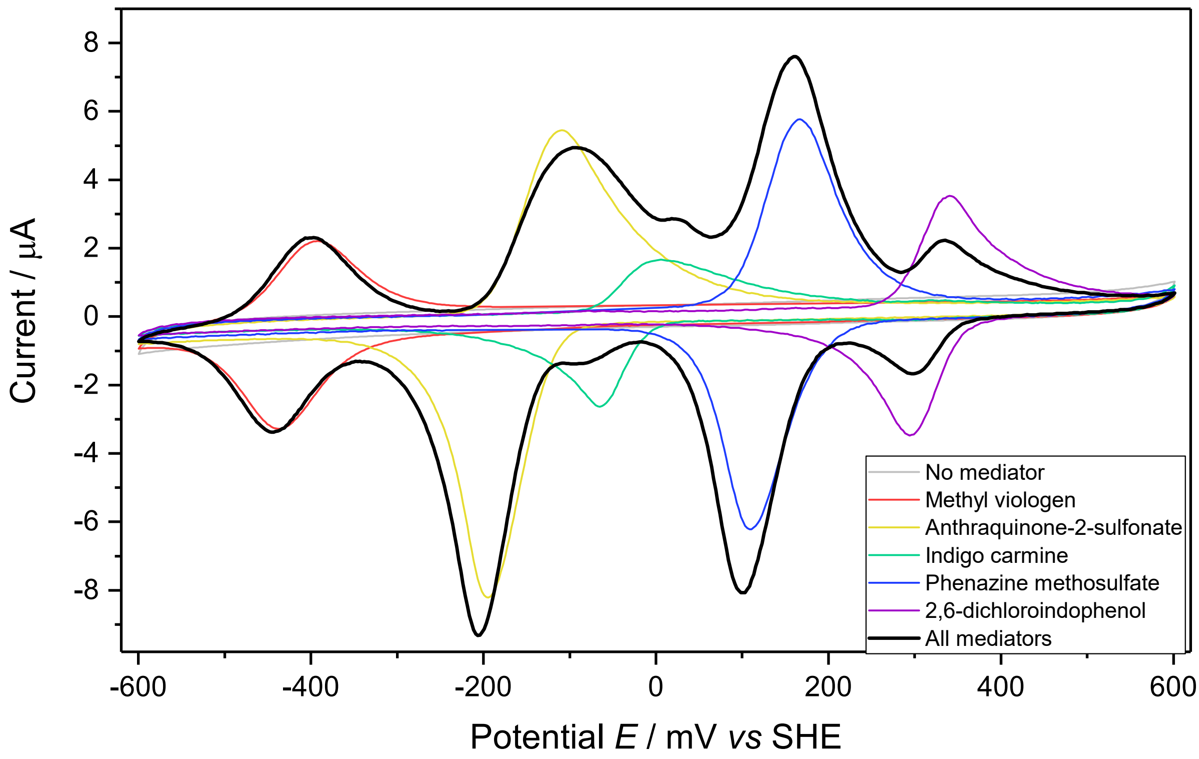

Figure S2. Cyclic voltammogram demonstrating electrochemical control within the infrared microspectroscopic-electrochemical cell. Recorded at a scan rate of 10 mV s^{-1}, with a background electrolyte of hydrogenase crystal stabilisation buffer (pH 5.9) additionally containing the redox mediators methyl viologen, anthraquinone-2-sulfonate, indigo carmine, phenazine methosulfate, and 2,6-dichloroindophenol (each at a concentration of 1 mM). See Table S1 for additional analysis.

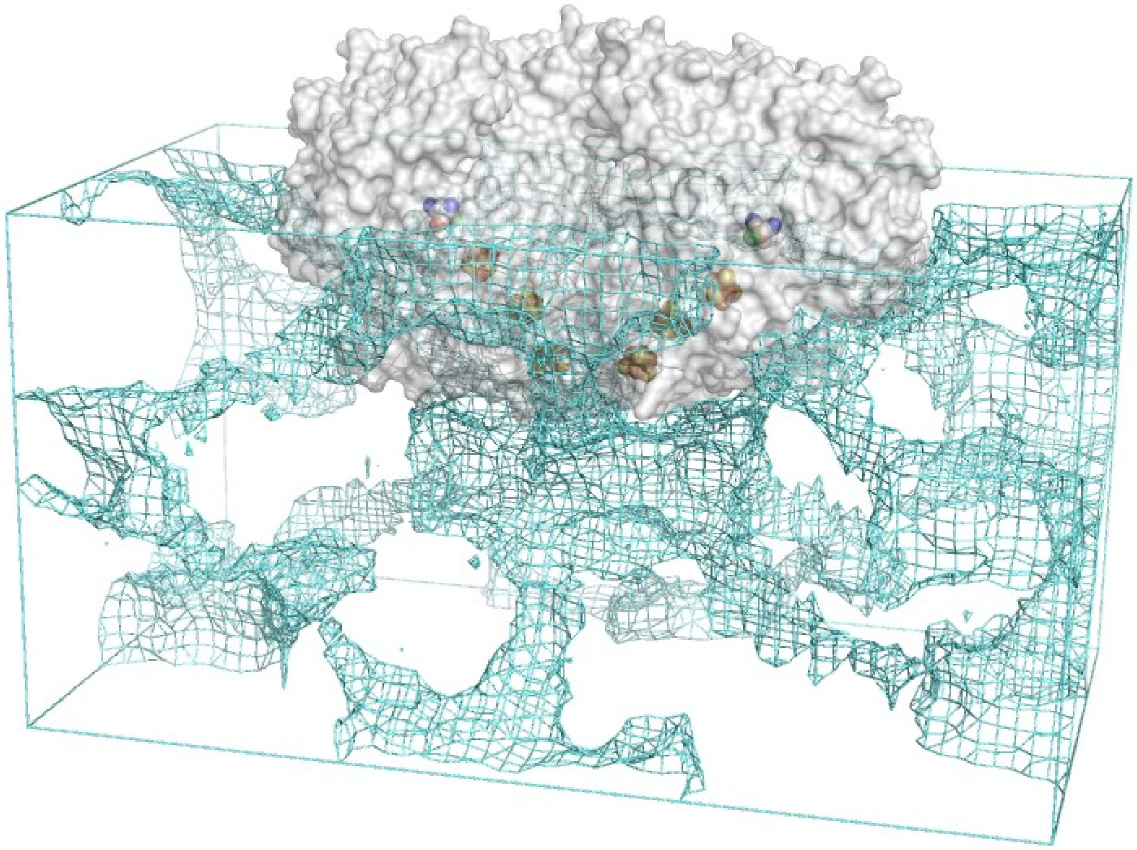

Figure S3. Illustration of the solvent channels within a crystal of Hyd1. Turquoise mesh represents the solvent channels (radii of 5.4-6.6 Å) between each dimer of heterodimers of Hyd1 within the crystal lattice. Redox mediators are able to diffuse into the crystal along the solvent channels to enable electrical contact between the distal FeS cluster and the working electrode.

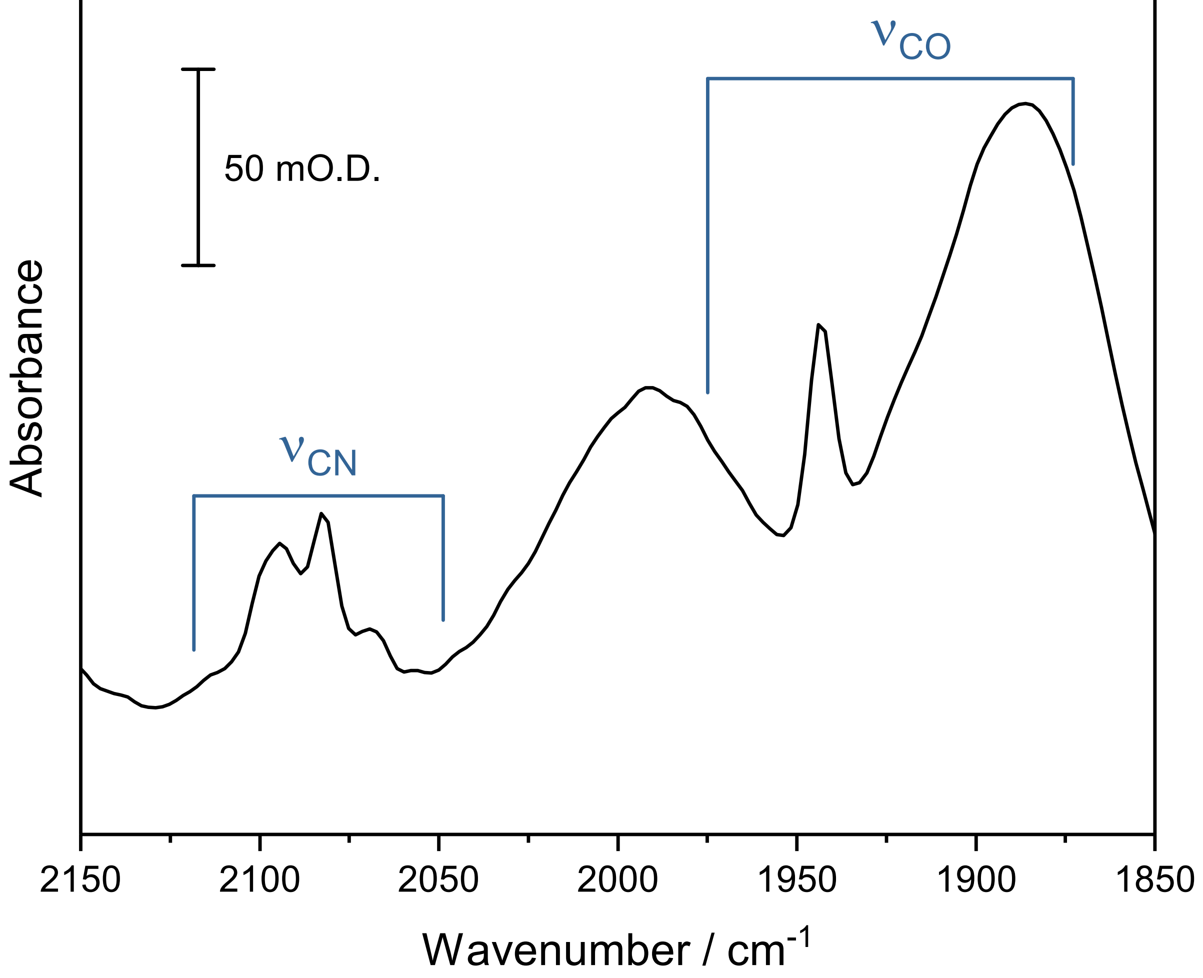

Figure S4. Raw IR spectrum of a single Hyd1 crystal at pH 5.9 before any electrochemical manipulation, recorded at the open circuit potential set by the oxidised mediator mixture (+209 mV). Regions where the ν_{CO} and ν_{CN} bands are observed are marked.

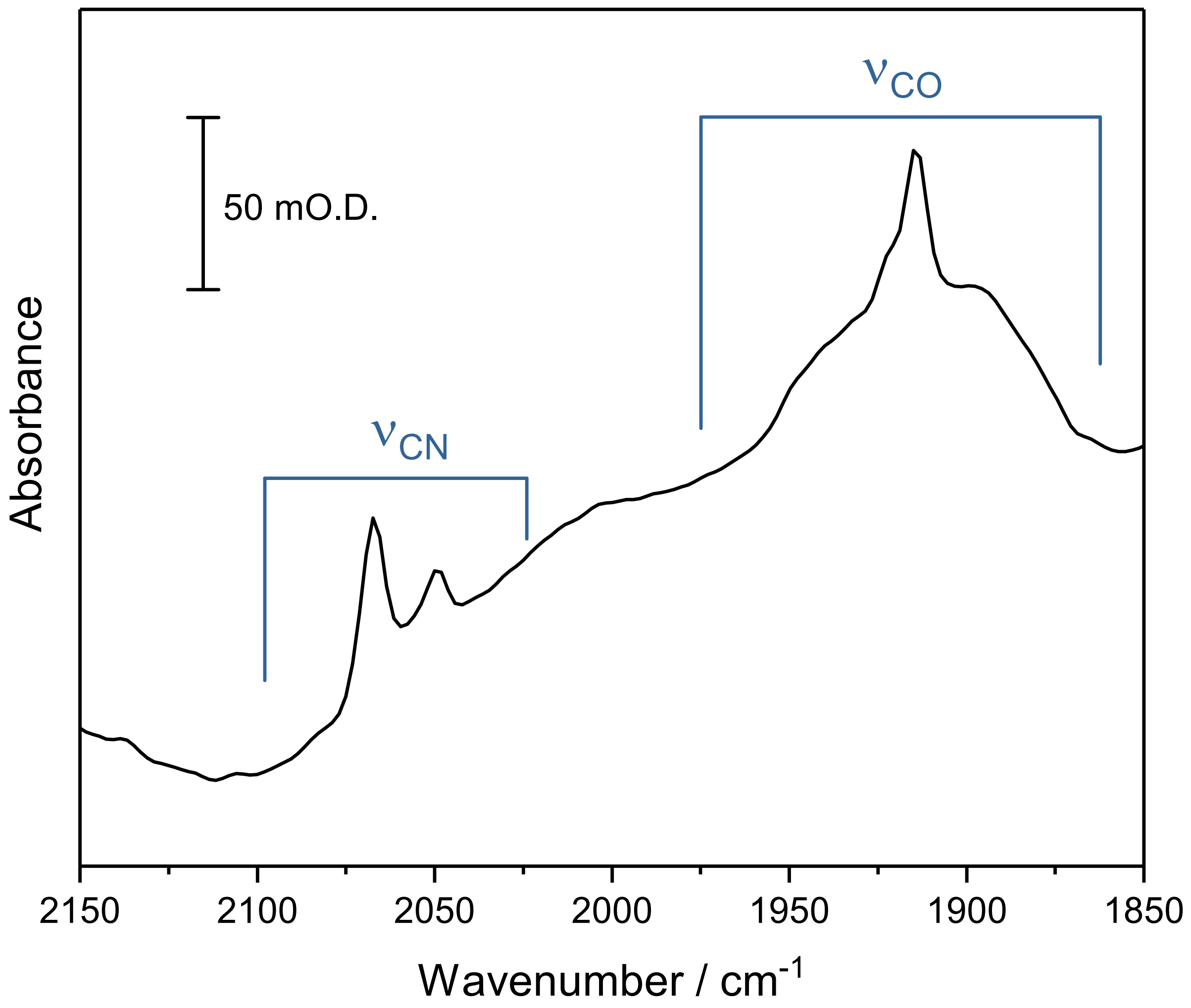

Figure S5. Raw IR spectrum of a single Hyd1 crystal at pH 5.9 poised at −597 mV. Spectrum recorded after electrochemical reduction for 1 hour. Regions where the ν_{CO} and ν_{CN} bands are observed are marked.

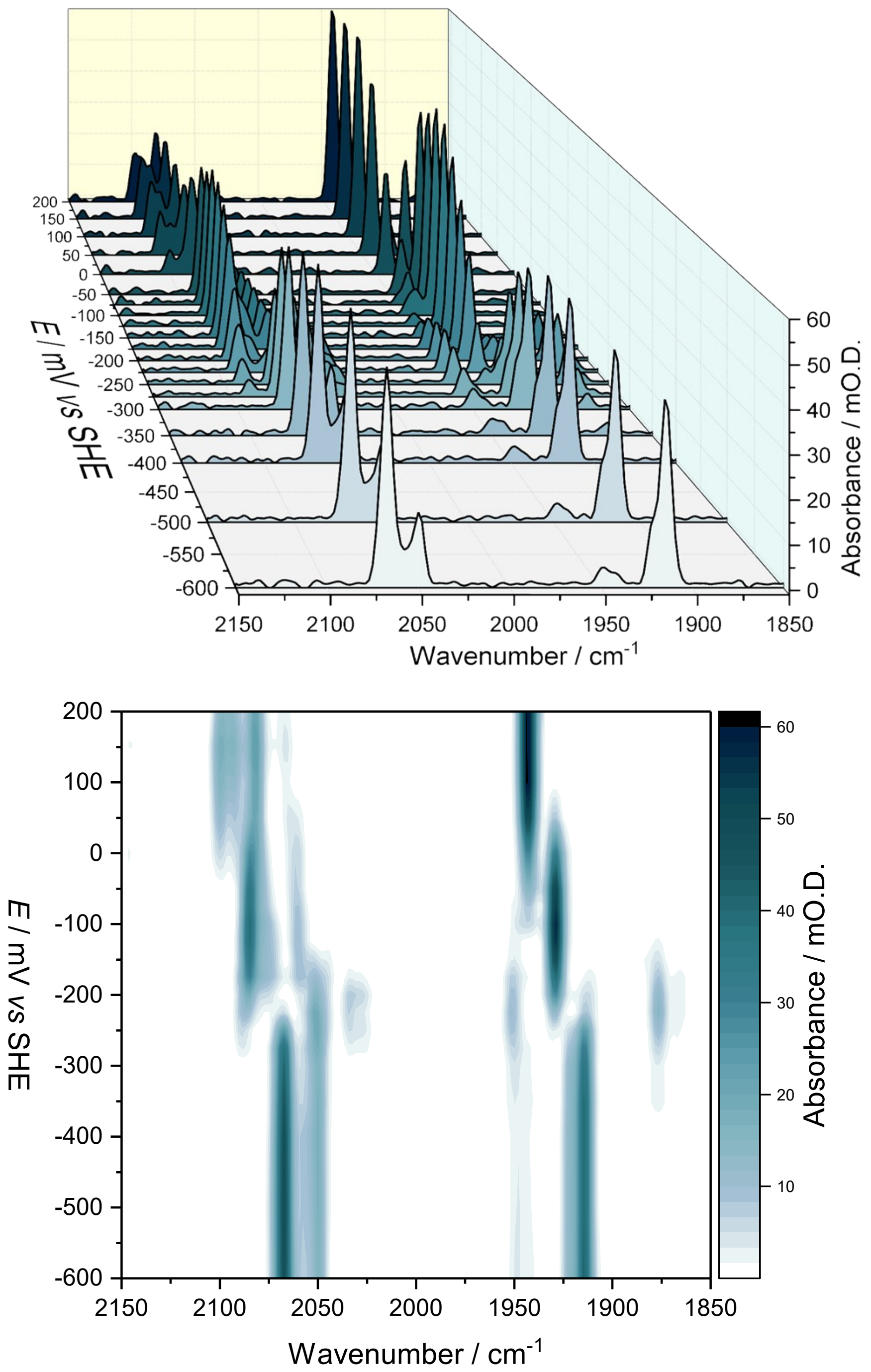

Figure S6. Electrochemical oxidative titration of a single crystal of Hyd1 at pH 5.9.

Top: Baseline corrected IR spectra of an electrochemical oxidative titration of a single crystal of Hyd1 at pH 5.9 recorded using the IR microspectroscopic-electrochemical technique showing the potential dependence of each active site redox species. The full ν_{CO} and ν_{CN} regions are shown.

Bottom: 'Heatmap' plot showing the potential dependence of each redox species for a single crystal of Hyd1 at pH 5.9. Changes in absorbance are shown by the change in colour intensity as indicated by the scale on the right.

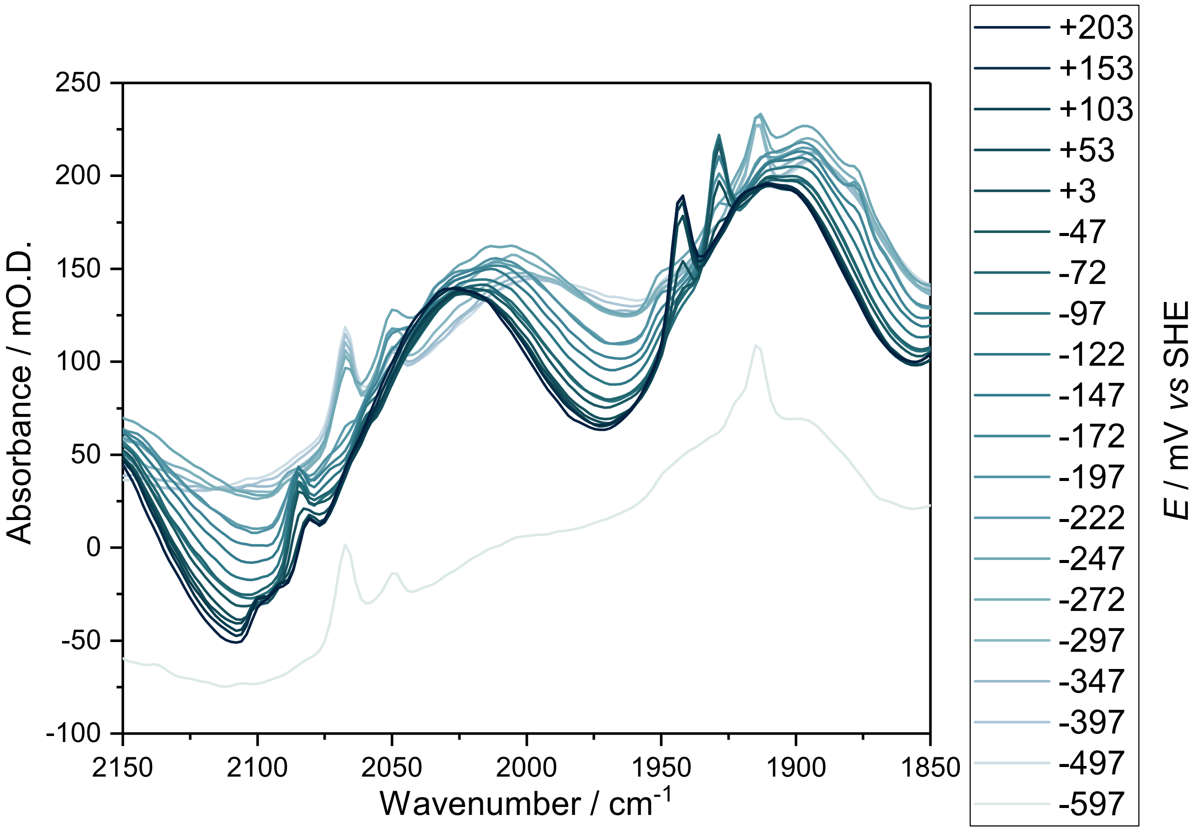

Figure S7. Raw IR spectra for pH 5.9 oxidative titration. Overlaid raw infrared spectra measured during an electrochemical oxidative titration of a single crystal of Hyd1 at pH 5.9. The IR spectrum at −597 mV is offset relative to the rest of the electrochemical titration due to collection of a new background spectrum. Note all spectra were recorded from the same 15 × 15 µm^2 area of the crystal. Baseline oscillations in the raw data are consistent with etalon fringes and a water layer of approximately 30 µm, consistent with the dimensions of the crystals and spacers used in this study.

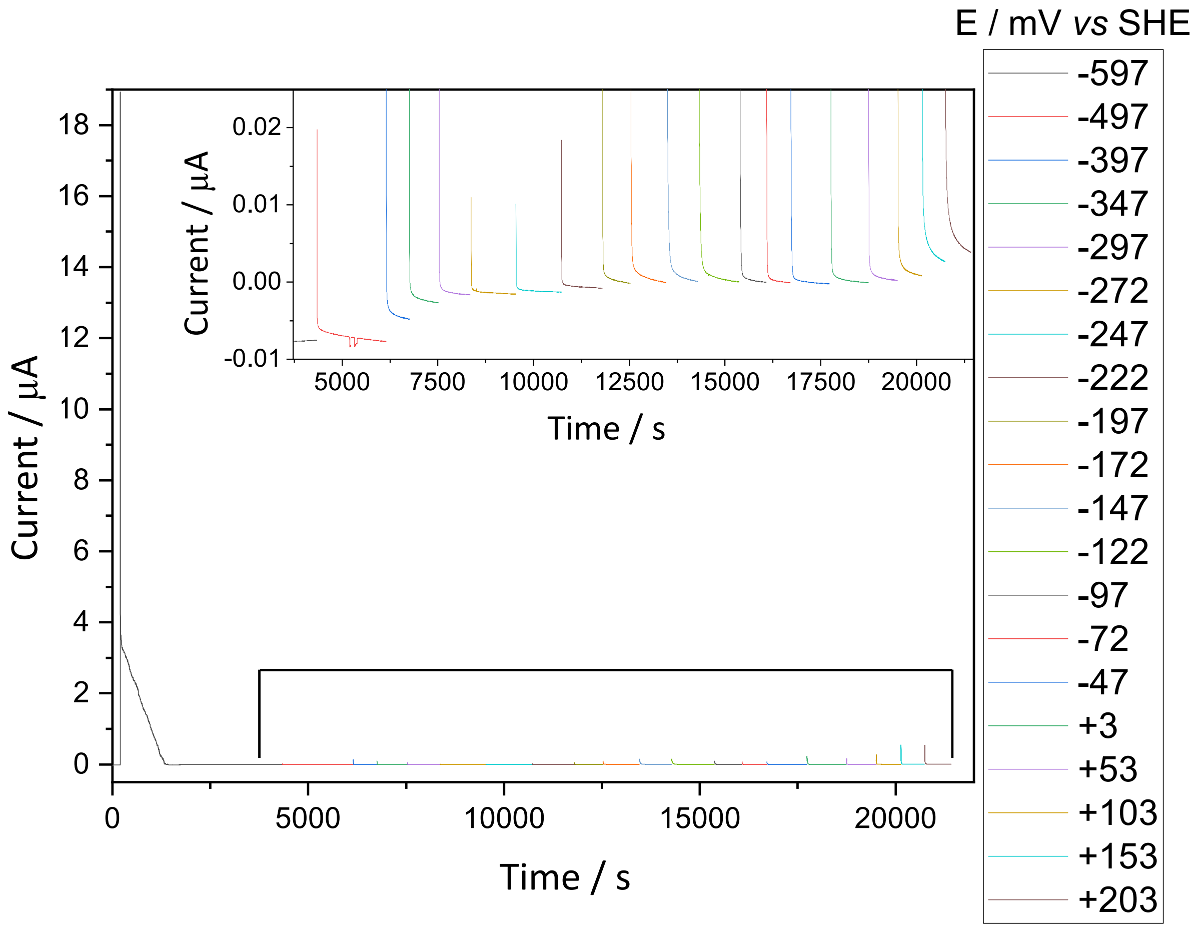

Figure S8. Chronoamperometry traces recorded during Hyd1 pH 5.9 microspectroscopic-electrochemical oxidative titration shown in Figure 2. A potential of −597 mV was applied for 3720 s prior to IR spectra being recorded. For the purposes of constructing the redox titration in Figure 3A of the main text, equilibrium was judged spectroscopically.

In general 'spectroscopic equilibrium' coincided with net zero current at most applied potentials. However at potentials more positive than +103 mV, or more negative than −347 mV, a small residual current (<|8 nA|) was observed. This small residual current is less than 0.5% of the peak current observed during voltammograms of the mediator cocktail in the microspectroscopic-electrochemical cell (Figure S2), and small residual currents are expected in thin-layer spectroscopic-electrochemical cells. Critically, however, we find that crystal position on the working electrode surface (i.e. whether it is near the centre or near the edge) has no discernible effect on the recorded redox titrations.

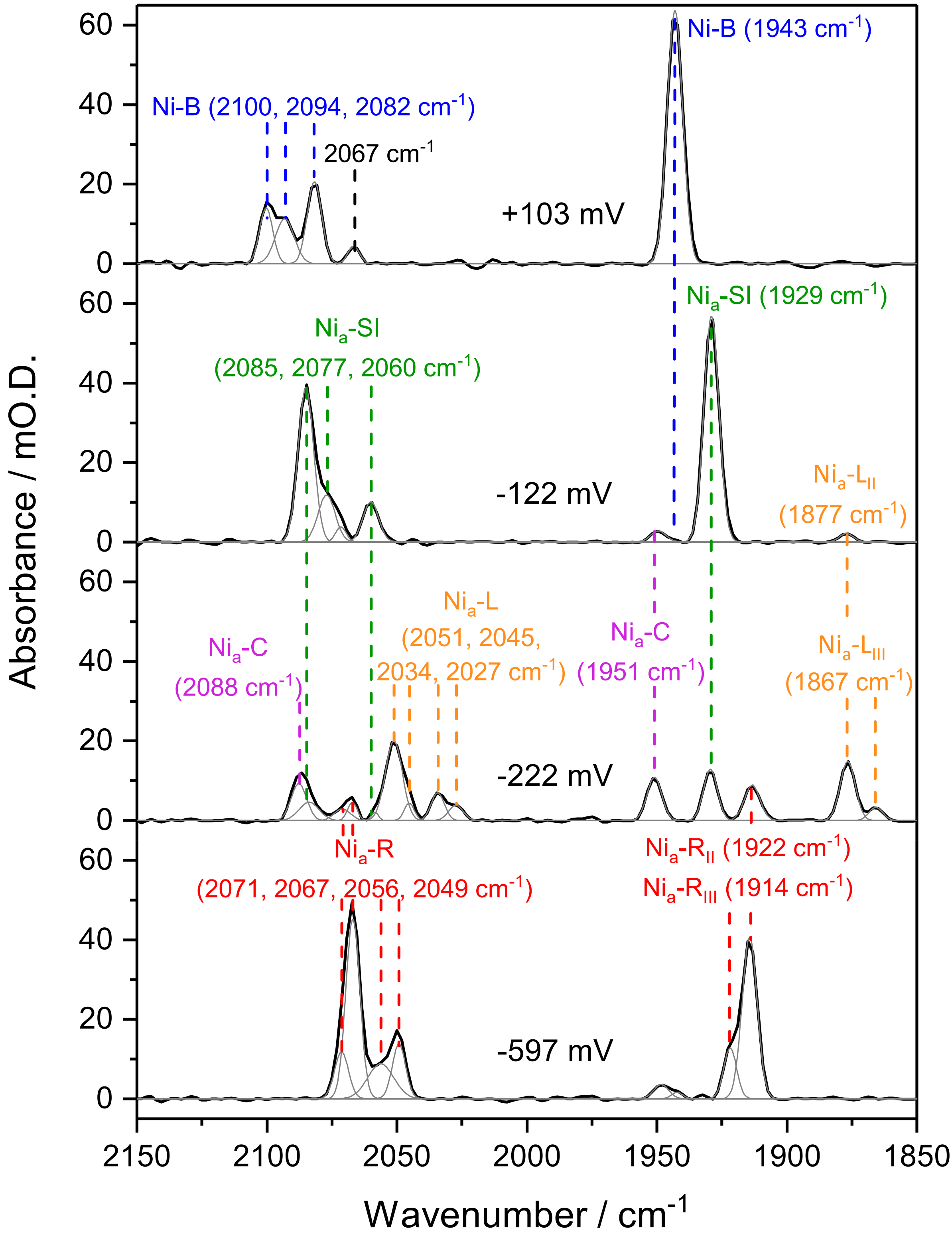

Figure S9. Representative potential-dependent spectra at pH 5.9. Infrared spectra of a single crystal of Hyd1, pH 5.9, poised at selected potentials as noted. The potentials selected reflect those at which maximum intensity for each active site redox species is observed during the redox titration shown in Figure 3A. Fitted peaks are shown in grey.

Table S2. Comparing active site species at pH 5.9. The relative ν_{CO} band positions of each active site species, to the nearest cm^{-1}, at pH 5.9 observed for Hyd1 in crystallo, in solution, or adsorbed on an electrode (PFIRE).

**Method**	**Position of ν_{CO} band centre / cm^{-1}**						
	Ni-B	Ni_a-SI	Ni_a-C	Ni_a-L_{II}	Ni_a-L_{III}	Ni_a-R_{II}	Ni_a-R_{III}
**In crystallo**[(a)]	1943	1929	1951	1877	1866	1922	1914
**Solution**[(a)]	1943	1929	1951	1877	1866	1922	1914
**PFIRE**[(b)]	1943	1929	1952	1877	1868	1922	1915

(a) This work

(b) Hidalgo *et al.* 2015[7]

We have reassigned the Ni_a-L absorbances relative to the original literature, as discussed in the main text, such that both the Ni_a-$L_{I, II, III}$ and Ni_a-$R_{I, II, III}$ sub-states are labelled in order of decreasing wavenumber of the active site CO stretch, ν_{CO}.

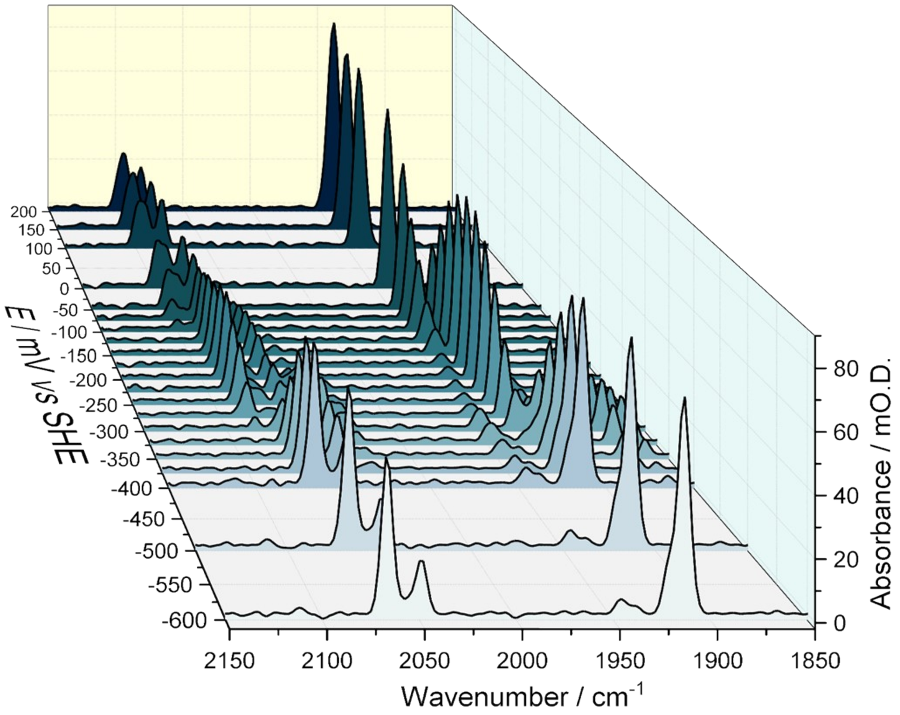

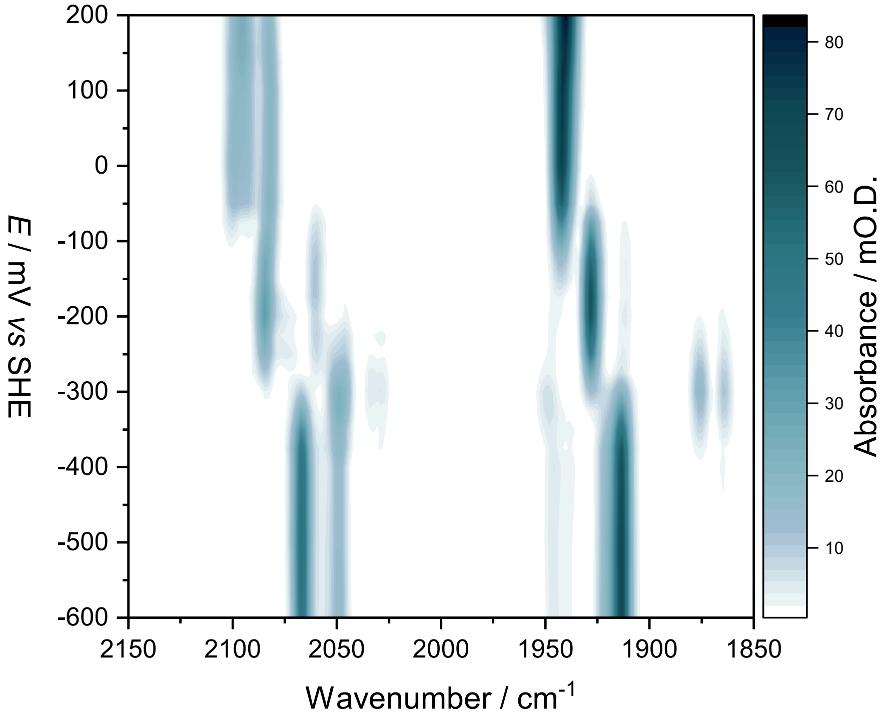

Figure S10. Electrochemical oxidative titration of a single crystal of Hyd1 at pH 8.0.
Top: Baseline corrected IR spectra of an electrochemical oxidative titration of a single crystal of Hyd1 at pH 8.0 recorded using the IR microspectroscopic-electrochemical technique showing the potential dependence of each active site redox species. The full ν_{CO} and ν_{CN} regions are shown.

Bottom: 'Heatmap' plot showing the potential dependence of each redox species for a single crystal of Hyd1 at pH 8.0. Changes in absorbance are shown by the change in colour intensity as indicated by the scale on the right.

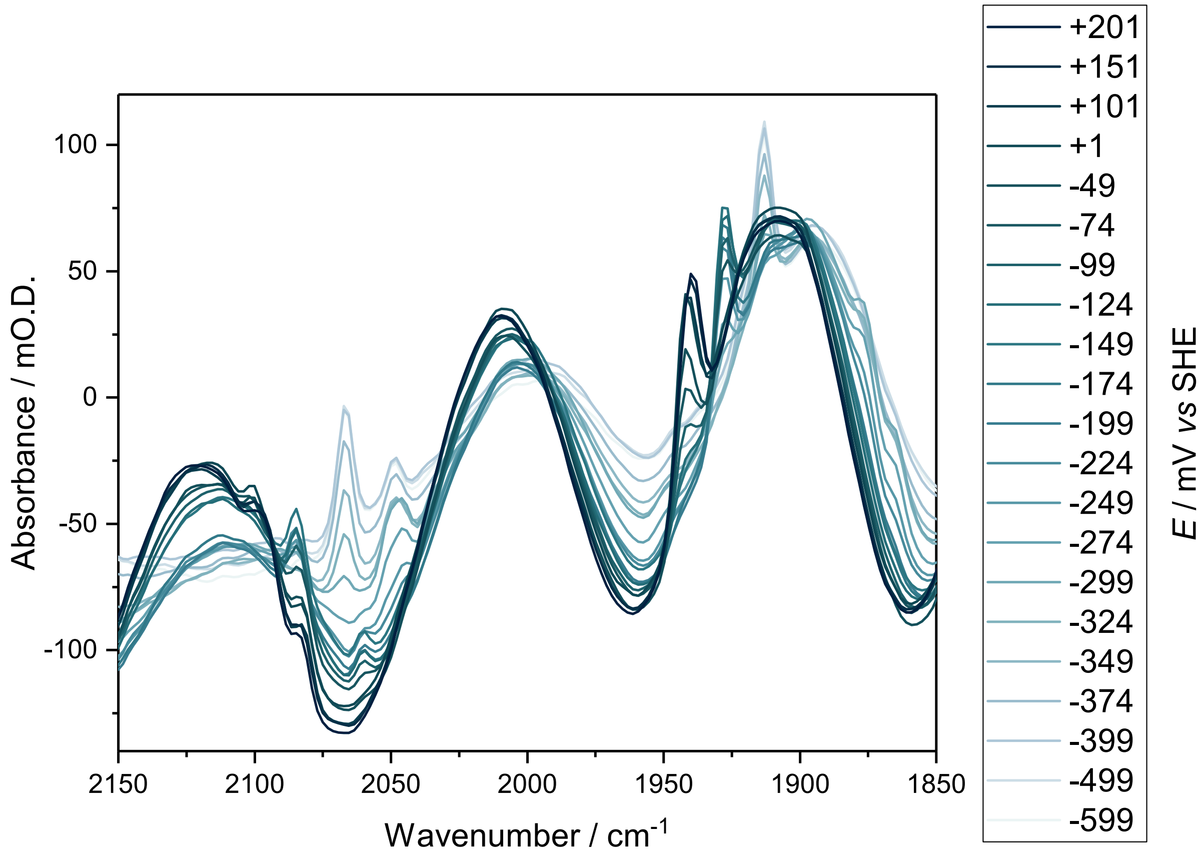

Figure S11. Raw IR spectra for pH 8.0 oxidative titration. Overlaid raw infrared spectra measured during an electrochemical oxidative titration of a single crystal of Hyd1 at pH 8.0. Note all spectra were recorded from the same 15 × 15 µm^2 area of the crystal.

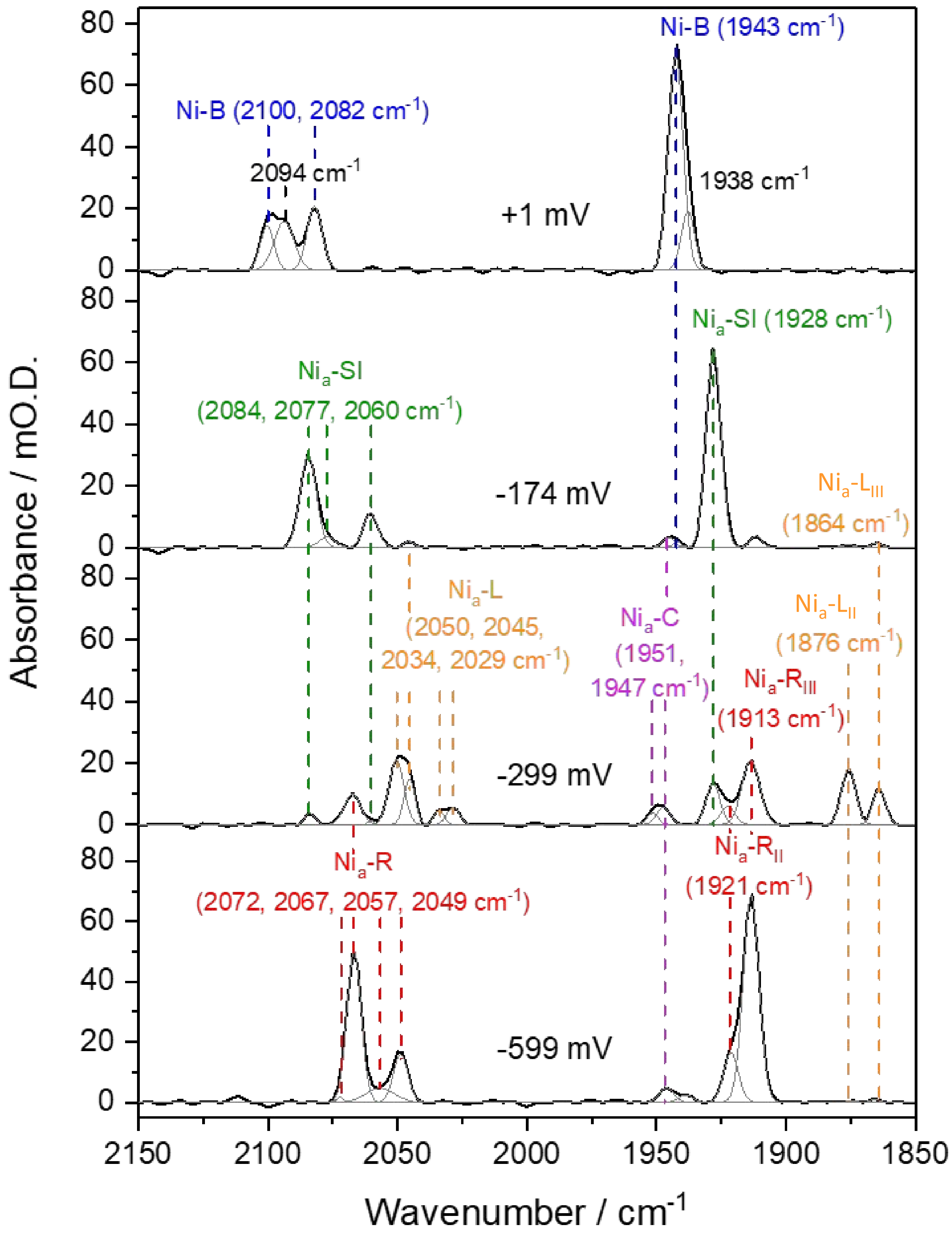

Figure S12. Representative potential-dependent spectra at pH 8.0. Infrared spectra of a single crystal of Hyd1, pH 8.0, poised at selected potentials as noted. The potentials selected reflect those at which maximum intensity for each active site redox species is observed during the redox titration shown in Figure 4. Fitted peaks are shown in grey.

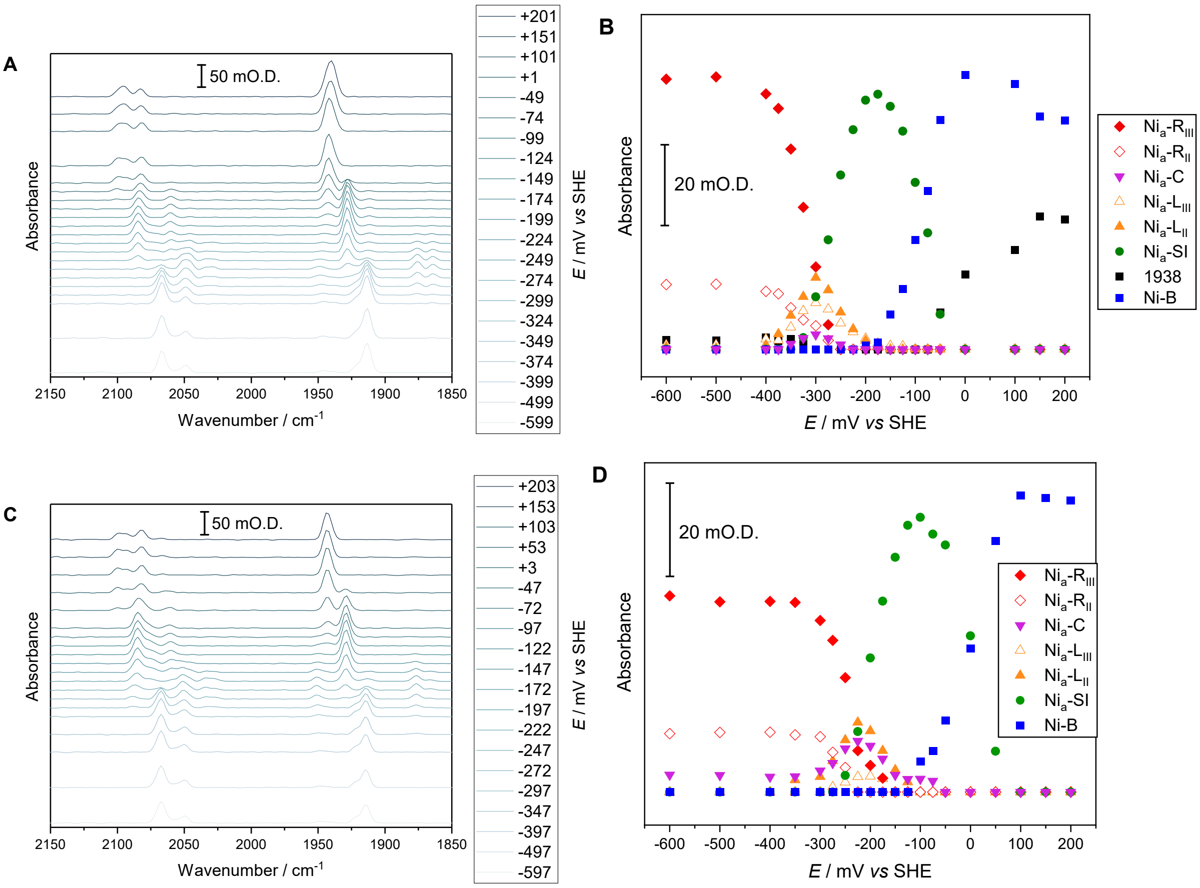

Figure S13. A comparison of pH 5.9 and pH 8.0. The electrochemical redox titrations of a single crystal of Hyd1 recorded using the IR microspectroscopic-electrochemical technique at pH 8.0 (A, B) and pH 5.9 (C, D). A: The baseline-corrected IR spectra showing the ν_{CO} and ν_{CN} regions across the potential range −600 to +200 mV collected at pH 8.0. B: A speciation plot illustrating potential dependence of all active site states at pH 8.0, including a species with ν_{CO} at 1938 cm^{-1}, discussed in the main text. C: The baseline-corrected IR spectra showing the ν_{CO} and ν_{CN} regions across the potential range −600 to +200 mV collected at pH 5.9. D: A speciation plot illustrating potential dependence of all active site states at pH 5.9.

Table S3. The pH dependence of active site ν_{CO}. The wavenumber positions of the ν_{CO} bands of the active site of Hyd1 crystals. The values are rounded to 1 decimal place from the fitted parameter.

pH	Position of ν_{CO} band / cm^{-1}							
	Ni-B	Ni-1938	Ni_a-SI	Ni_a-C	Ni_a-L_{II}	Ni_a-L_{III}	Ni_a-R_{II}	Ni_a-R_{III}
**5.9**	1943.0	-	1929.0	1951.0	1876.6	1865.8	1922.1	1914.3
**8.0**	1942.4	1938.2	1928.0	1951.5<br>1947.4	1875.1	1864.1	1921.2	1913.5

Band positions, reported the nearest 0.1 cm^{-1}, are derived from Gaussian profiles fitted to baseline-corrected IR spectra with the aid of 2nd derivative spectra.

We have reassigned the Nia-L absorbances relative to the original literature, as discussed in the main text, such that both the Ni_a-$L_{I,\ II,\ III}$ and Ni_a-$R_{I,\ II,\ III}$ sub-states are labelled in order of decreasing wavenumber of the active site CO stretch, *v*CO.

Table S4. Proximal cluster potentials. Potentials of the 5+/4+ and 4+/3+ transitions of the proximal cluster of Hyd1 compared to other O_2-tolerant NiFe hydrogenases.

Redox transition	Proximal cluster midpoint potential / mV				
	Native Hyd1 pH 6[16]	Native Hyd1 pH 7[27]	Hyd1 medial cluster P242C variant pH 6[16]	*Aquifex aeolicus* pH 7.4[28]	*R. eutropha* H16[28, a]
5+/4+	230 ± 30	211 ± 15	175 ± 15	232 ± 20	160
4+/3+	3 ± 30	4 ± 15	90 ± 20	87 ± 20	−60

(a) Data from *R. eutropha* H16 are reproduced as originally reported in reference 26

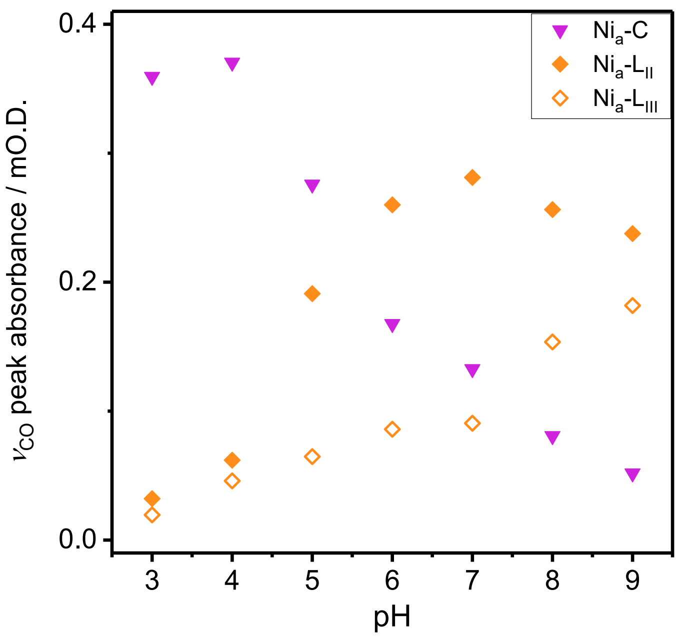

Figure S14. The relative populations of Ni_a-C, Ni_a-L_{II}, and Ni_a-L_{III} are pH dependent**.** Peak absorbance data reproduced from PFIRE spectra, recorded as a function of pH at potentials at which the total [Ni_a-C + Ni_a-L] is optimised. The pH-dependence observed in PFIRE data is in good agreement with the pH dependence observed in single crystals shown in Figure 5 of the main text. The Ni_a-L sub-states observed in *E. coli* Hyd1 do not share the same pH dependence, implying multiple proton acceptor sites.

We have reassigned the Ni_a-L absorbances relative to the original literature, as discussed in the main text, such that both the Ni_a-$L_{I,\ II,\ III}$ and Ni_a-$R_{I,\ II,\ III}$ sub-states are labelled in order of decreasing wavenumber of the active site CO stretch, *v*CO.

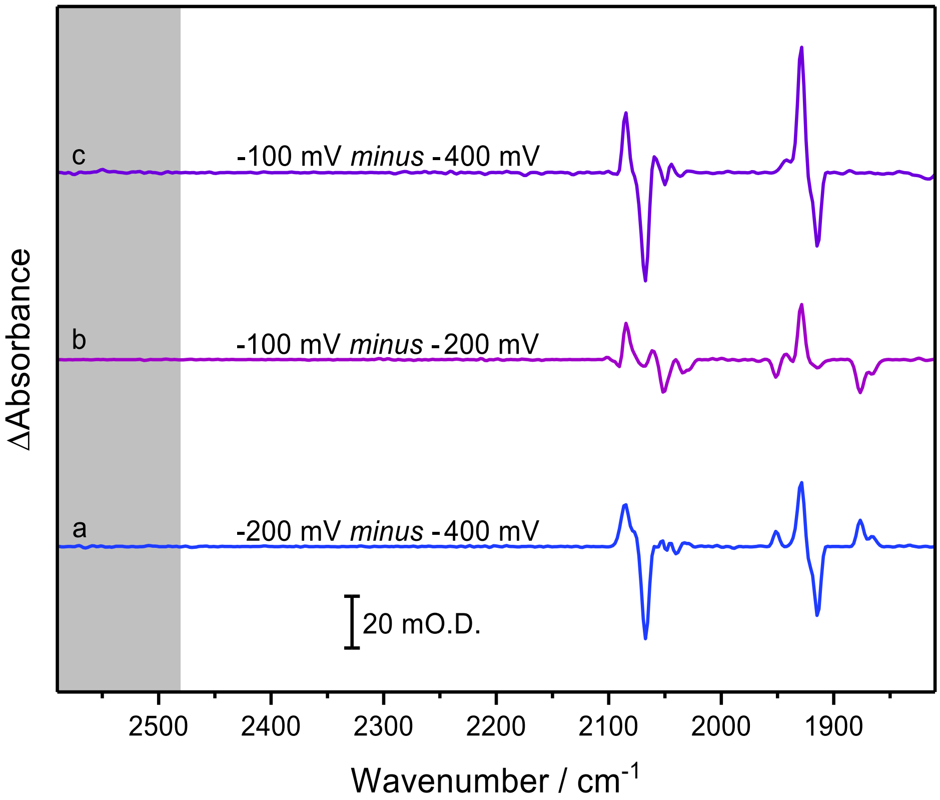

Figure S15. Single crystal difference spectra show no evidence of cysteine-S protonation in either Ni_a-$R_{II/III}$ or Ni_a-$L_{II/III}$. Difference spectra were calculated from raw single crystal microspectroscopy data recorded at pH 5.9 and reported in Figure S7: **a** shows the Ni_a-C/Ni_a-L redox level *minus* the Ni_a-R redox level; **b** shows the Ni_a-SI redox level *minus* the Ni_a-C/Ni_a-L redox level; **c** shows the Ni_a-SI redox level *minus* the Ni_a-R redox level. The approximate S-H stretching region (*v*SH) is shaded in grey. There is no evidence for a change in cysteine-S protonation at any redox level, which implies either the existence of S-H in all redox states or the absence of S-H in all redox states. Note that in Hyd1 crystals we do not observe the Ni_a-L_I state, for which Tai *et al.* have reported *v*SH at 2505 cm^{-1} in the [NiFe] hydrogenase from *D. vulgaris* Miyazaki F (*Dv*MF).[15] Tai *et al.* additionally noted that cysteine-S protonation was not observed in either the Ni_a-SI or Ni_a-C states of *Dv*MF. Our data therefore suggests that there are no changes in cysteine-S protonation between any of the Ni_a-SI, Ni_a-C, Ni_a-$L_{II/III}$, or Ni_a-$R_{II/III}$ states in Hyd1. In combination with evidence from collected IR data which suggests that a terminal cysteine-S ligand becomes deprotonated during the transition from Ni_a-L_I to Ni_a-$L_{II/III}$, and from Ni_a-R_I Ni_a-$R_{II/III}$,[26] these data provide further evidence that terminal cysteine-S is not protonated in the Ni_a-$L_{II/III}$ or Ni_a-$R_{II/III}$ states.

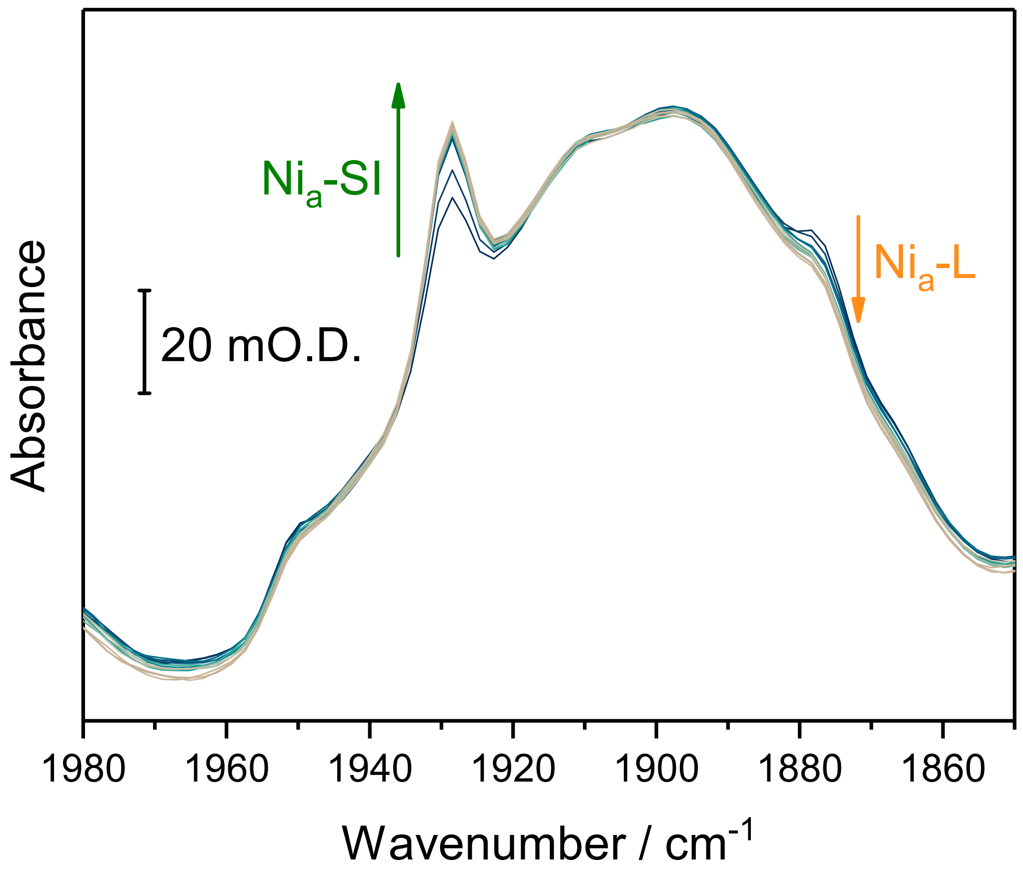

Figure S16. The transition of Ni$_a$-L to Ni$_a$-SI in single crystals of Hyd1 at pH 5.9 during an oxidative potential step from −197 mV to −172 mV. Raw overlaid IR spectra are shown, with baseline corrected spectra in Figure S17.

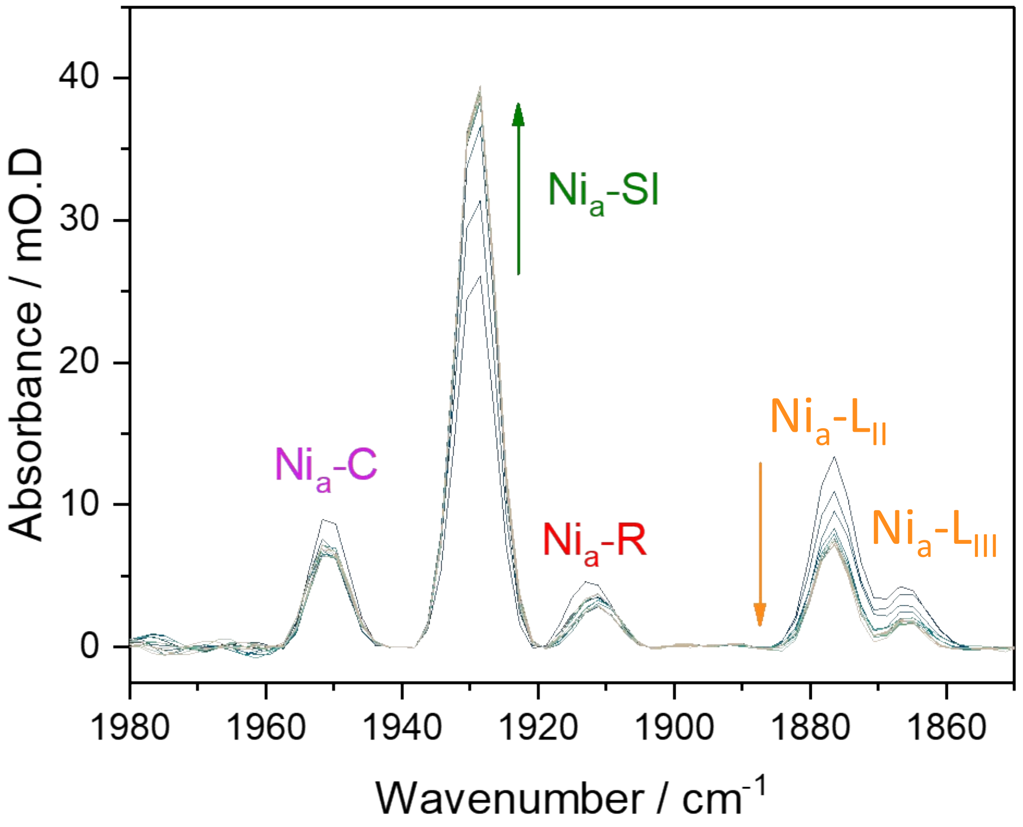

Figure S17. The transition of Ni_a-L to Ni_a-SI in a single crystal of Hyd1 at pH 5.9 during an oxidative potential step from −197 mV to −172 mV. Baseline corrected IR spectra are shown, for raw IR spectra see Figure S16.

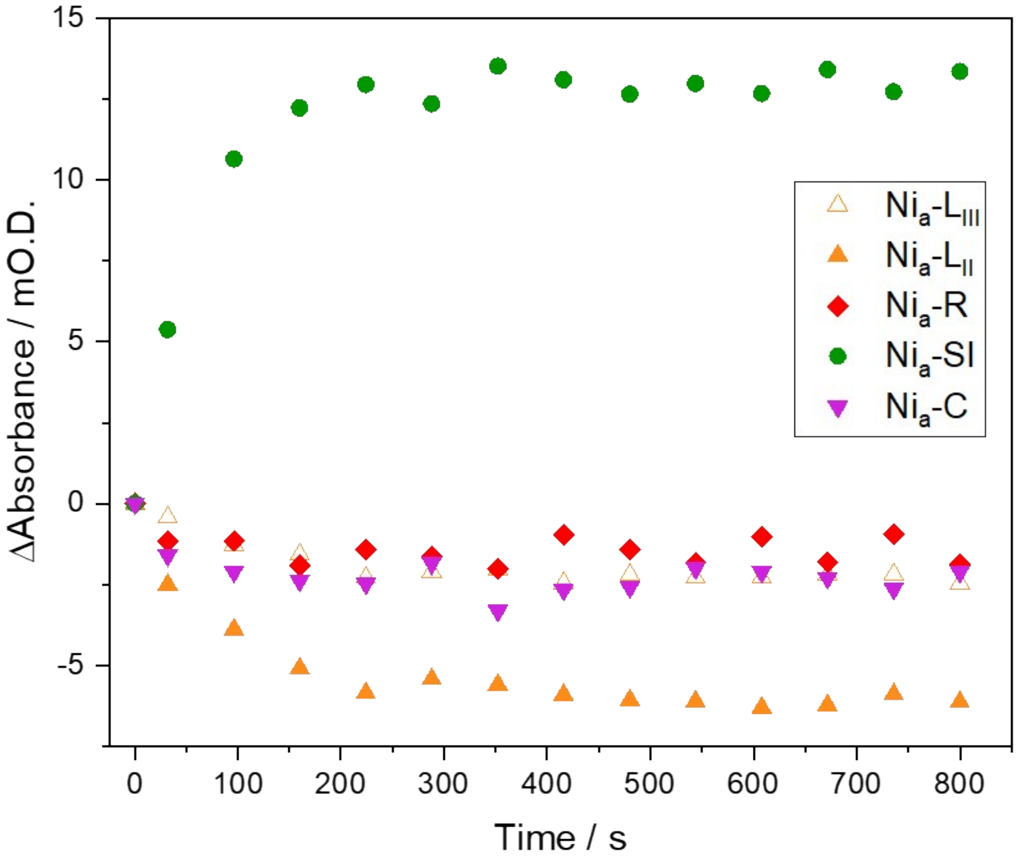

Figure S18. Time dependence of the active site redox species in a single crystal of Hyd1 at pH 5.9, following an oxidative potential step from −197 mV to −172 mV. These are the same data as reported in Figure 6B, but here also include the Ni$_a$-R and Ni$_a$-C species, whose intensities remain approximately constant after collection of the first spectrum following the potential step (ca 32 s). The Ni$_a$-C data was subject to 10 % fitting error; the Ni$_a$-R data were subject to 10 % fitting error. Qualitatively similar results were obtained from multiple crystals.

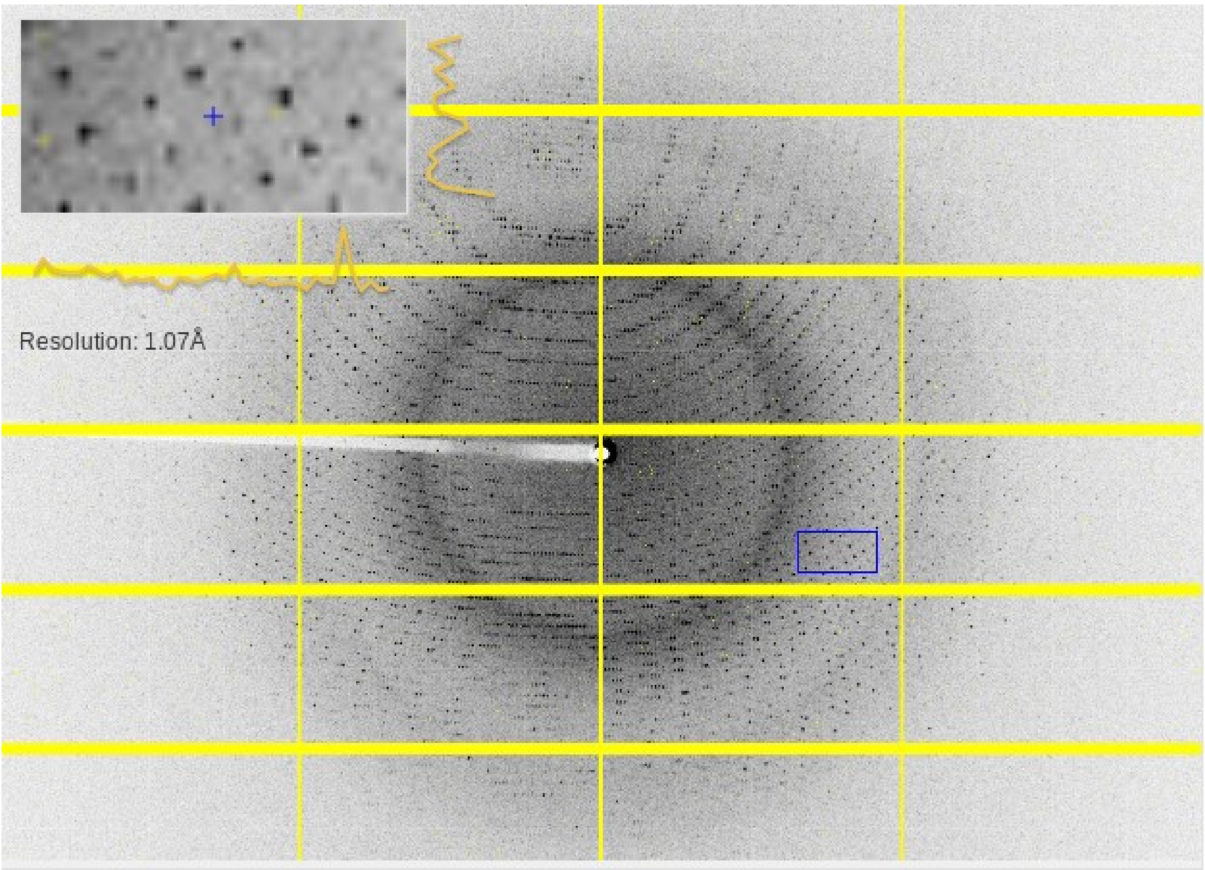

Figure S19. X-ray diffraction data for mediator soaked Hyd1 crystals. X-ray diffraction image collected from Hyd1 crystal after extensive (6hr) soaking in redox mediator cocktail, demonstrating crystals still diffract X-rays to high resolution after prolonged exposure to redox mediators.

Table S5. X-ray data collection statistics for Hyd1 crystals soaked in mediator cocktail for 6 hours.

Sample	Hyd1 + Mediator cocktail
**Space group**	$P2_12_12_1$
**Unit cell dimensions (Å)**	a=94.23, b=98.12, c=183.13
**Resolution (Å)**	67.98–1.30 (1.32–1.30)
**Total reflections**	2,990,605 (111,583)
**Unique reflections**	407,014 (18,647)
**Completeness (%)**	98.2 (91.0)
**Multiplicity**	7.3 (6.0)
**<I/σ>**	10.7 (1.0)
**R_{merge} (%)**	9.7 (187)
**R_{pim} (%)**	3.5 (82.7)
**$CC_{1/2}$**	0.997 (0.372)

Values are parenthesis refer to data in the highest resolution shell.

Supplementary References

1 Z. Y. Zhou and S. G. Sun, *Electrochim. Acta*, 2005, **50**, 5163–5171.

2 S. M. Rosendahl, F. Borondics, T. E. May, T. M. Pedersen and I. J. Burgess, *Rev. Sci. Instrum.*, 2011, 82, 083105.

3 P. A. Ash, H. A. Reeve, J. Quinson, R. Hidalgo, T. Zhu, I. J. McPherson, M. W. Chung, A. J. Healy, S. Nayak, T. H. Lonsdale, K. Wehbe, C. S. Kelley, M. D. Frogley, G. Cinque and K. A. Vincent, *Anal. Chem.*, 2016, **88**, 6666–6671.

4 P. A. Ash, S. B. Carr, H. A. Reeve, A. Skorupskaite, J. S. Rowbotham, R. Shutt, M. D. Frogley, R. M. Evans, G. Cinque, F. A. Armstrong and K. A. Vincent, *Chem. Commun.*, 2017, **53**, 5858–5861.

5 A. J. Healy, P. A. Ash, O. Lenz and K. A. Vincent, *Phys. Chem. Chem. Phys.*, 2013, **15**, 7055–7059.

6 P. Paengnakorn, P. A. Ash, S. Shaw, K. Danyal, T. Chen, D. R. Dean, L. C. Seefeldt and K. A. Vincent, *Chem. Sci.*, 2017, **8**, 1500–1505.

7 R. Hidalgo, P. A. Ash, A. J. Healy and K. A. Vincent, *Angew. Chemie*, 2015, **127**, 7216–7219.

8 A. J. Bard and L. R. Faulkner, *Electrochemical Methods: Fundamentals and Applications*, Wiley, New York, 2001.

9 H. Ogata, K. Nishikawa and W. Lubitz, *Nature*, 2015, **520**, 571–574.

10 R. M. Evans, E. J. Brooke, S. A. M. Wehlin, E. Nomerotskaia, F. Sargent, S. B. Carr, S. E. V. Phillips and F. A. Armstrong, *Nat. Chem. Biol.*, 2016, **12**, 46–50.

11 B. L. Greene, G. E. Vansuch, C. H. Wu, M. W. W. Adams and R. B. Dyer, *J. Am. Chem. Soc.*, 2016, **138**, 13013–13021.

12 B. L. Greene, C. H. Wu, G. E. Vansuch, M. W. W. Adams and R. B. Dyer, *Biochemistry*, 2016, **55**, 1813–1825.

13 E. J. Brooke, R. M. Evans, S. T. A. Islam, G. M. Roberts, S. A. M. Wehlin, S. B. Carr, S. E. V. Phillips and F. A. Armstrong, *Biochemistry*, 2017, **56**, 132–142.

14 R. M. Evans, P. A. Ash, S. E. Beaton, E. J. Brooke, K. A. Vincent, S. B. Carr and F. A. Armstrong, *J. Am. Chem. Soc.*, 2018, **140**, 10208–10220.

15 H. Tai, K. Nishikawa, Y. Higuchi, Z. Mao and S. Hirota, *Angew. Chemie*, 2019, **131**, 13419–13424.

16 M. M. Roessler, R. M. Evans, R. A. Davies, J. Harmer and F. A. Armstrong, *J. Am. Chem. Soc.*, 2012, **134**, 15581–15594.

17 B. J. Murphy, R. Hidalgo, M. M. Roessler, R. M. Evans, P. A. Ash, W. K. Myers, K. A. Vincent and F. A. Armstrong, *J. Am. Chem. Soc.*, 2015, **137**, 8484–8489.

18 H. Tai, K. Nishikawa, M. Suzuki, Y. Higuchi and S. Hirota, *Angew. Chemie - Int. Ed.*, 2014, **53**, 13817–13820.

19 S. Kumar and S. K. Acharya, *Anal. Biochem.*, 1999, **268**, 89–93.

20 O. S. Ksenzhek, S. A. Petrova and M. V. Kolodyazhny, *Bioelectrochemistry Bioenerg.*, 1977, **4**, 346–357.

21 P. G. Tratnyek, T. E. Reilkoff, A. W. Lemon, M. M. Scherer, B. A. Balko, L. M. Feik and B. D.

Henegar, 2001, **6**, 172–179.

22 A. D. Broadbent and R. J. Melanson, *Can. J. Chem*, 1975, **53**, 3757–3760.

23 R. Cammack, V. M. Fernandez and E. Claude Hatchikian, *Methods Enzymol.*, 1994, **243**, 43–68.

24 W. N. Lanzilotta and L. C. Seefeldt, *Biochemistry*, 1997, **36**, 12976–12983.

25 C. L. Bird and A. T. Kuhn, *Chem. Soc. Rev.*, 1981, **10**, 49.

26 P. A. Ash, R. Hidalgo and K. A. Vincent, *ACS Catal.*, 2017, **7**, 2471–2485.

27 H. Adamson, M. Robinson, J. J. Wright, L. A. Flanagan, J. Walton, D. Elton, D. J. Gavaghan, A. M. Bond, M. M. Roessler and A. Parkin, *J. Am. Chem. Soc.*, 2017, **139**, 10677–10686.

28 M. E. Pandelia, W. Nitschke, P. Infossi, M. T. Giudici-Orticoni, E. Bill and W. Lubitz, *Proc. Natl. Acad. Sci. U. S. A.*, 2011, **108**, 6097–6102.
